# Fundamentals and Applications of Dual‐Frequency Magnetic Particle Spectroscopy: Review for Biomedicine and Materials Characterization

**DOI:** 10.1002/advs.202416838

**Published:** 2025-02-22

**Authors:** Hans‐Joachim Krause, Ulrich M. Engelmann

**Affiliations:** ^1^ Institute of Biological Information Processing Bioelectronics (IBI‐3) Forschungszentrum Jülich 52425 Jülich Germany; ^2^ Medical Engineering and Applied Mathematics FH Aachen University of Applied Sciences 52428 Jülich Germany

**Keywords:** magnetic biosensing, magnetic fluid hyperthermia (MFH), magnetic frequency mixing detection (FMMD), magnetic immunoassays, magnetic particle imaging (MPI), magnetic particle spectroscopy (MPS), micromagnetic simulation

## Abstract

Superparamagnetic nanoparticles (MNP) offer exciting applications for engineering and biomedicine in imaging, diagnostics, and therapy upon magnetic excitation. Specifically, if excited at two distinct frequencies *f*_1_ and *f*_2_, MNP responds with magnetic intermodulation frequencies *m*·*f*_1_ ± *n*·*f*_2_ caused by their nonlinear magnetization. These mixing frequencies are highly specific for MNP properties, uniquely characterizing their presence. In this review, the fundamentals of frequency mixing magnetic detection (FMMD) as a special case of magnetic particle spectroscopy (MPS) are reviewed, elaborating its functional principle that enables a large dynamic range of detection of MNP. Mathematical descriptions derived from Langevin modeling and micromagnetic Monte‐Carlo simulations show matching predictions. The latest applications of FMMD in nanomaterials characterization as well as diagnostic and therapeutic biomedicine are highlighted: analysis of the phase of the FMMD signal characterizes the magnetic relaxation of MNP, allowing to determine hydrodynamic size and binding state. Variation of excitation amplitudes or magnetic offset fields enables determining the size distribution of the particles’ magnetic cores. This permits multiplex detection of polydisperse MNP in magnetic immunoassays, realized successfully for various biomolecular targets such as viruses, bacteria, proteins, and toxins. A portable magnetic reader enables portable immunodetection at point‐of‐care. Future applications toward theranostics are summarized and elaborated.

## Graphical abstract

Superparamagnetic nanoparticles show non‐linear magnetization behavior. Thus, they respond with magnetic intermodulation frequencies upon dual‐frequency magnetic excitation. These uniquely characterize their hydrodynamic and core sizes as well as binding states. This enables exciting applications in engineering and biomedicine, foremost as magnetic immunodetection for diagnostic point‐of‐care services as well as complementary techniques to magnetic particle imaging and adjunct therapy inspiring theranostics.

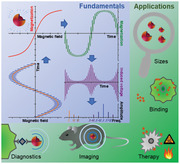

## 1 Introduction

Over the past two decades, the use of magnetic nanoparticles (MNP) has largely increased due to their small size that offers unique magnetic properties as well as versatile applicability in materials science and biomedicine.^[^ ^1^, ^2^, ^3^, ^4^, ^5^ ^]^ On the one hand, MNP are small enough to infiltrate into (sub‐)microscale structures and navigate inside biological systems.^[^ ^6^, ^7^ ^]^ On the other hand, they display superparamagnetic characteristics (e.g., for sizes below ≈30 nm for magnetite^[^ ^8^ ^]^). Such superparamagnetic nanoparticles possess single net magnetic moments, wherefore an ensemble of MNP (at room temperature) behaves like a paramagnet but with a manyfold higher magnetization comparable to ferromagnets, henceforth coining the term *superpara*magnetic.^[^ ^9^ ^]^ This allows non‐hysteretic, non‐remanent magnetic manipulation of these MNP in static magnetic fields^[^ ^10^, ^11^ ^]^ or characteristic identification of their nonlinear magnetization relaxation dynamics when exposed to an alternating magnetic field (AMF).^[^ ^12^, ^13^ ^]^

Although the usage of static magnetic fields on MNP already enabled many applications of MNP, e.g., in magnetic separation^[^ ^14^, ^15^ ^]^ and (drug) delivery/transportation techniques,^[^ ^16^, ^17^, ^18^ ^]^ as optical filters^[^ ^19^ ^]^ and sensing systems for, e.g., temperature and pH,^[^ ^20^ ^]^ detecting and interpreting the relaxation dynamics of MNP in an AMF even expands the capabilities of MNP as it generally allows to conclude on the MNP intrinsic properties themselves that generate the particle response. In other words, understanding the frequency‐dependent MNP response to a periodic excitation AMF additionally allows localization, quantification, and characterization of MNP.^[^ ^21^, ^22^, ^23^ ^]^ The technique is there therefore commonly named magnetic particle *spectroscopy* (MPS). Indeed, MPS has become the go‐to‐choice for quickly assessing relaxation behavior of an ensemble of MNP experimentally due to its easy and versatile applicability, sensitivity, and non‐destructive method. Here, a sinusoidal excitation field is applied to a MNP sample and the particle signal is received as a dynamic voltage proportional to the excitation field‐derivative of the MNP magnetization.^[^ ^24^, ^25^ ^]^ From this, the particle signal is typically derived and interpreted as the harmonic spectrum in the frequency domain. Since MPS employs frequencies in the kilohertz‐regime to which the MNP responds within milli‐ to microseconds, consequently, it delivers quasi real‐time information and results.^[^ ^26^, ^27^ ^]^ This renders MPS‐based techniques especially attractive as sensors in materials science and probes in the biomedical sector. For example, it is commonly applied in materials characterization to quantify the iron concentration of a given MNP sample^[^ ^28^, ^29^ ^]^ and has been extensively used to determine changes in MNP arrangement, microstructure, immobilization of MNP, and viscosity effects.^[^ ^30^, ^31^, ^32^ ^]^ Some prominent examples of MPS‐based sensitive probing of biomedical nanostructures are the detection of antibodies, viruses, and molecules demonstrated in vitro.^[^ ^33^, ^34^, ^35^ ^]^ The application of MPS in conjunction with other MNP relaxation dynamics‐based techniques such as heating agents, imaging tracers, or biosensing markers for the advancement biomedical applications is of rising importance currently.^[^ ^36^, ^37^, ^38^, ^39^ ^]^

The sensitivity and versatility of MPS can be further increase by adding another AMF thereby introducing the frequency mixing magnetic particle detection (FMMD) method: Applying a dual‐frequency AMF consisting of a low‐frequency (high‐amplitude, ≈ 10 kHz) magnetic driving field simultaneously with a high‐frequency (low‐amplitude, ≈ 1 kHz) magnetic excitation field to MNP induces a multi‐harmonic spectrum with intermodulation frequencies.^[^ ^40^, ^41^ ^]^ The response of MNP to the applied AMF is governed by the particles’ dynamic nonlinear magnetization, which can be indirectly measured as the output voltage of pick‐up‐coils used in a FMMD setup,^[^ ^42^ ^]^ which is fundamentally described by the particle relaxation processes that drive the magnetic moments of MNP. FMMD detects harmonics (like MPS) and additional intermodulation products specific to the dual‐frequency magnetic excitation, which arise from the characteristic nonlinear magnetization response of MNP to the applied field. FMMD allows exclusive detection of the (intermodulated) sum frequency components that result from the frequency mixing and adds uniquely characteristic information about the MNP sample under investigation.

Intermodulation frequency mixing terms are the sum of integer multiples, *m* · *f*_1_  +  *n* · *f*_2_, with m,n∈Z, of the original input frequencies *f*_1_ and *f*_2_, superimposed on the received signal. Such additional mixing frequency terms are a general phenomenon of a nonlinear system's response to a two (or more) frequency excitation. Depending on the application, it is either detrimental or amplifies the desired signal. For example, in audio processing it is commonly perceived as an undesired perturbation^[^ ^43^ ^]^ as well as in microwave systems for (early 1980s) mobile telecommunication systems.^[^ ^44^ ^]^ However, special cases also allow beneficial applications of frequency mixing for frequency amplifiers or selection techniques to improve the efficiency of nonlinear wireless systems^[^ ^45^ ^]^ and nonlinear optics.^[^ ^46^ ^]^ More recent examples are opto‐electronic frequency amplification in graphene for GHz‐applications in telecommunication and radio/light detection^[^ ^47^ ^]^ and two‐frequency modulation in dynamic atomic force microscopy for successful extraction of Morse forces.^[^ ^48^ ^]^

As this review will show, FMMD also uses purposefully induced intermodulation as a beneficial method for analyzing the magnetic response of MNP. Its frequency mixing terms are particularly sensitive for MNP detection compared to classical magnetic characterization techniques such as vibrating sample magnetometer (VSM) or superconducting quantum interference devices (SQUID) that measure the bulk magnetization as well as innovative common single frequency applications such as magnetic particle spectroscopy (MPS) and magnetic particle imaging (MPI) (see Section 4.1 for details). This fact gives rise to specific applications of FMMD in biomedicine and materials characterization, as highlighted throughout this review in Chapter 4.

The MNP relaxation dynamics in AMF forms the origin of the all applications mentioned above and the ability for its prediction and interpretation are the pacemakers for MNP‐based technological advancement in biomedical applications. Therefore, extensive efforts have been made to describe the nonlinear magnetization response of MNP in an AMF mathematically and physically. The first simple but robust models succeeded in describing the overall magnetization of an ensemble of MNP with the Langevin function,^[^ ^11^, ^49^ ^]^ from which the average magnetic core size of the ensemble can be derived.^[^ ^50^, ^51^ ^]^ Also, a simplistic estimation of the ensemble's heating potential can be calculated based on linear magnetization by approximating the ensemble relaxation time by combining intrinsic Néel relaxation and external Brownian relaxation mechanisms^[^ ^52^ ^]^ (sub section 2.1.2 for details). However, these approaches are non‐realistic because they neglect both nonlinearity of the magnetic response of MNP as well as non‐equilibrium conditions, which are important for MNP excitation in the kilohertz‐regime.^[^ ^26^ ^]^ One step further, the 2‐level‐approximation describes the nonlinearity of the MNP dynamic response from a generalized probability for a unidirectional reversal process of individual particles.^[^ ^13^ ^]^ It was successfully applied to predict key dependencies of external field and intrinsic particle‐properties on magnetic particle heating^[^ ^53^, ^54^ ^]^ and expanded for Brownian rotation.^[^ ^55^, ^56^ ^]^ Nevertheless, it ultimately lacks accuracy since the actual motion of individual particles on the microscale is driven by thermal fluctuations and therefore described best by a stochastic process combining Néel‐Brownian relaxation and thermal fluctuations in a stochastic calculus approach.^[^ ^12^, ^57^ ^]^ In chapter 3 of this review, we further discuss approaches on modeling the dynamic magnetization of dual frequency FMMD of MNP for spectroscopic applications in more detail.

In this work, we comprehensively summarize the state‐of‐the‐art fundamentals mathematically describing the MPS and FMMD signal (i.e., the MNP magnetization during excitation) and elaborate how MPS and FMMD measurements can be set‐up, modified, and analyzed by applying this knowledge. We therefore first present the background on MNP, MPS, and FMMD necessary to understand the signal generation in chapter 2. Subsequently, we describe different approaches to mathematically model the FMMD signal generation and apply these to interpret and analyze the dependencies of the FMMD signal generation in chapter 3, as a foundation for its applications. Chapter 4 consequently offers an overview of the current application status of MPS‐based techniques in general and the advancement of the FMMD method in specific, ranging from biosensing and combined diagnostics (Section 4.1) to advanced nanoparticle materials characterization (Section 4.2). Finally, the last chapter concludes and summarizes our contribution, which aims to advance the fundamental understanding of FMMD to inspire more research in the research community.

## 2 Fundamentals of FMMD

### 2.1 Magnetization of Superparamagnetic Nanoparticles

If any material is exposed to a magnetic field, ***H***, the material responds by acquiring a magnetization, ***M***. This magnetization originates from aligning the individual atomic magnetic moments, ***μ***, within the material and is defined per unit volume, *V*, as $M\;=\frac{1}{V}\;\sum_{n=1}^{N}\sum \mu_{n}$. For most materials, the magnetization scales linearly with the magnetic field according to

(1) M=χ·H

with magnetic susceptibility, ***χ***. Note that ***χ*** is generally a 3D tensor, which can be described by the partial derivatives of the magnetization components, *M_i_* with respect to the field components, *H_j_* ^[^ ^9^ ^]^ as

(2) $$\chi_{ij}=\frac{\partial M_{i}}{\partial H_{j}}\;$$

This term reduces to a scalar only for homogeneous and isotropic materials so that Equation (1) holds. The full magnetic response of materials to an applied field, *H*, is given by the magnetic induction.

(3) $$B\;=\mu_{0}\;\left(H+M\right)$$

where μ_0_ is the vacuum permeability (also dented as magnetic constant) *µ*_0_ = 4π·10^−7^ Vs/Am.

In contrast to Equation (1), which is valid for general materials, the response of ferromagnets to sufficiently strong applied magnetic fields is nonlinear and of high magnetization (several orders of magnitude higher than non‐ferromagnetic materials).

On a microscopic level, a ferromagnet actually consists of many, typically micrometer‐sized areas of uniformly aligned magnetic moments, denoted as magnetic domains.^[^ ^58^ ^]^ Within a single domain, the exchange interaction between neighboring spins aligns the atomic magnetic moments parallel to each other. The orientation of magnetization with a single domain originates from the energetic competition between exchange interaction, magnetic anisotropy, and the magnetic stray field (further details can be found in refs. [1, 9, 59]). Since the exchange interaction is isotropic in space, the direction of magnetization within a single domain is exclusively determined by anisotropy energy, while a magnetic stray field builds up only externally that aligns with the neighboring magnetic domain orientations. These anisotropically preferred directions of magnetization within a crystal, i.e., those directions, where anisotropy energy is minimal, are denoted easy axes.

Magnetic nanoparticles (MNP) are typically assumed to be spherically shaped^[^ ^1^, ^60^ ^]^ and consist of magnetic matter in monocrystalline arrangement of sizes ranging between 5 and 25 nm. The atoms that form an individual particle are arranged in an epitaxial, crystallographically ordered structure, which is even visible with high‐resolution TEM imaging.^[^ ^61^, ^62^ ^]^ Due to this order, their atomic magnetic moments strongly interact, just as the spins in ferromagnets, and thereby display ferromagnetic behavior. However, due to their small nanometer size, MNP falls below the magnetic domain size limit (below which it is energetically not efficient anymore to compartmentalize in domains; e.g., below ≈100 nm for magnetite, iron‐platinum or nickel^[^ ^27^ ^]^) to uphold ferromagnetic behavior. For MNP, the magnetic anisotropy barrier can be approximated by

(4) $$\Delta E=K_{\mathrm{eff}}\;\cdot V_{C}$$

With the effective anisotropy constant, *K*_eff_, (summarizing all contributions to anisotropy energy, as explained above) and the particle's magnetic core volume, $V_{C}=\frac{\pi }{6}\;d_{C}^{3}$, with the core size (diameter), *d_C_*. In this state, the magnetic (anisotropy) energy barrier of the MNP (smaller than approx. 30nm in size) is smaller than the thermal energy, and the MNP cannot maintain a collective magnetic order among each other (but nevertheless the magnetization within each individual particle remains intact).^[^ ^27^ ^]^ I.e., *E*_therm_ ≈ *k_B_* · *T* > Δ*E* and the net magnetization of a sufficiently large ensemble of MNP is therefore zero and the ensemble of MNP is denoted as *thermalized*. MNP in this state are denoted as superparamagnetic and do not show ferromagnetic hysteresis. This behavior of magnetization can be well described mathematically using the Langevin function, as will be explained in the following Section 2.2.

**Figure 1:**
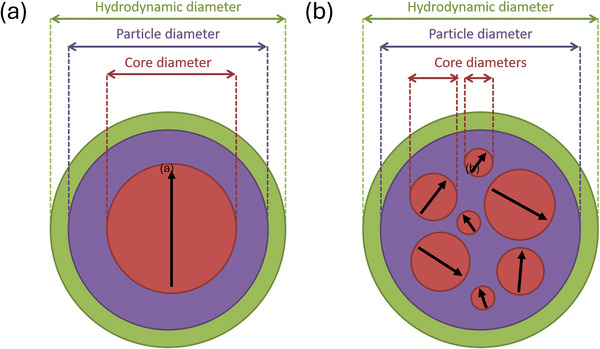
a) Illustration of a magnetic nanoparticle, consisting of a single magnetic core (e.g., magnetite) and an organic shell to yield the particle hydrophilic. In an aqueous solution, water molecules aggregate at the particle surface, additionally contributing to the overall hydrodynamic diameter. b) Multi‐core particle for which the organic shell contains several magnetic cores of different sizes.

A typical material for MNP is iron‐oxide magnetite (chemical formula: Fe_3_O_4_), which is frequently used for medical applications of MNP as it is generally biocompatible^[^ ^27^, ^60^ ^]^ and well‐tolerated by the human body.^[^ ^63^ ^]^ Another requirement from MNP in application is colloidal stability and adjustable magnetic properties,^[^ ^64^ ^]^ usually achieved by making MNP hydrophilic by surrounding the magnetic Fe_3_O_4_‐core with an organic shell, see **Figure** **1**a.

When submerged in aqueous solution, further molecules (ligands) settling at the shell will further increase the effective particle size, constituting the hydrodynamic size (diameter), *d_H_* = *d_C_*  + 2 · *d*_shell_ + 2 · *d*_coating_, with the thickness of the organic shell, *d*_shell_, and the thickness of the ligand coating, *d*_coating_, cf. Figure 1a. The magnetization of an MNP is majorly dependent on its core material; e.g., a magnetite particle consisting of, e.g., 10^5^ formula units magnetite (that is three iron and four oxygen atoms) exhibits a strong effective magnetic moment *m_p_* of the order of 3.8  ×  10^5^ *µ_B_*, with *µ_B_* = *e*ℏ/2*m_e_* = 9.274 × 10^−24^ Am^2^ denoting Bohr's magneton, the natural unit for expressing the magnetic moment of an electron. With this, a particle moment is much larger than any atomic magnetic moment (at most, an atomic moment sums up to 8 *µ_B_*, which is found in the case of Gadolinium^[^ ^65^ ^]^), i.e., *m_p_* ≫ μ and the term *super* in superparamagnetism refers to this strong magnetic response while the magnetization curve follows that of paramagnets^[^ ^9^, ^49^ ^]^ (cf. Langevin function, Equation (6) below). A superparamagnetic particle's magnetic moment is then described by the saturation magnetization and its core volume or size, respectively:

(5) $$m_{p}=M_{S}\;\cdot \;V_{C}=M_{S}\;\frac{\pi \cdot d_{C}^{3}}{6}$$

Due to synthesis processing, the shell can also contain multiple cores^[^ ^66^, ^67^ ^]^ as shown in Figure 1b. These multi‐core MNP^[^ ^68^ ^]^ show promising effects throughout all biomedical applications of MNP^[^ ^69^ ^]^ but at the same time add additional complexity to exact physical and magnetic MNP characterization,^[^ ^70^ ^]^ as their magnetic cores will strongly interact with each other^[^ ^71^ ^]^ and are certainly of deviating sizes and positionings (i.e., possess an intra‐core size‐distribution besides the inter‐particle size distribution).^[^ ^72^ ^]^ Indeed, multi‐core MNP imposes another level of challenges to describing MNP properties mathematically correctly.^[^ ^66^, ^72^, ^73^ ^]^ They are, however, an emerging entity for biomedical applications, and their uses and beneficial effects will be discussed in chapter 5. As in this review, we aim for a fundamental understanding and comprehensive mathematical description of the underlying principles leading to FMMD signal generation, multi‐core MNP is excluded in the following modeling and mathematical descriptions in chapter 3. However, the description of polydisperse MNP samples, developed in chapter 3.2 and applied consequently throughout the materials analysis and applications presented in this work henceforth, allows for a realistic prediction of real‐world samples.

#### 2.1.1 Non‐Dynamic Magnetization: Description in Thermal Equilibrium with the Langevin Function

Let us consider an ensemble of magnetic single‐domain particles that all have the same size (a so‐called monodisperse ensemble) in a volume *V*. Furthermore, the particles shall be non‐interacting, which is true if the particle concentration is low enough (e.g., Nanomag‐D SPIO (micromod GmbH, Rostock, Germany) with concentration *c*  =  4.3  ×  10^15^ particles per cubic cemtimeter^[^ ^74^ ^]^). Each individual particle contributes a maximum saturation magnetic moment *m_p_* of the order of 10^5^ *µ_B_* (see section 2.1 above). In thermodynamic equilibrium, the orientation of the particles’ moments, ***m**_p_*, is always assumed to be parallel to the applied field, ***H***, i.e., that there is an instantaneous reaction of the magnetization to a change in the field (i.e., there is no phase lag between ***M*** and ***H*** or in other words, no relaxation process of ***M*** allowed). At a given temperature, *T* and an applied field, ***H***, the average moment density of an ensemble of MNP is thus governed by the Boltzmann distribution with respect to ***H***. The average moment density in the direction of the field then represents the magnetic moment per volume, i.e., the magnetization ***M*** of the ensemble of magnetic particles^[^ ^9^ ^]^ as described above. The magnetization of an ensemble of non‐interacting and homogeneously distributed MNP in thermodynamic equilibrium then follows as (***M*** aligned to ***H***, therefore not written as a vector quantity)^[^ ^75^ ^]^

(6) $$M\left(H\right)=M_{s}\;\cdot L\left(\frac{m_{p}\mu_{0}H}{k_{B}T}\right).$$

**Figure 2:**
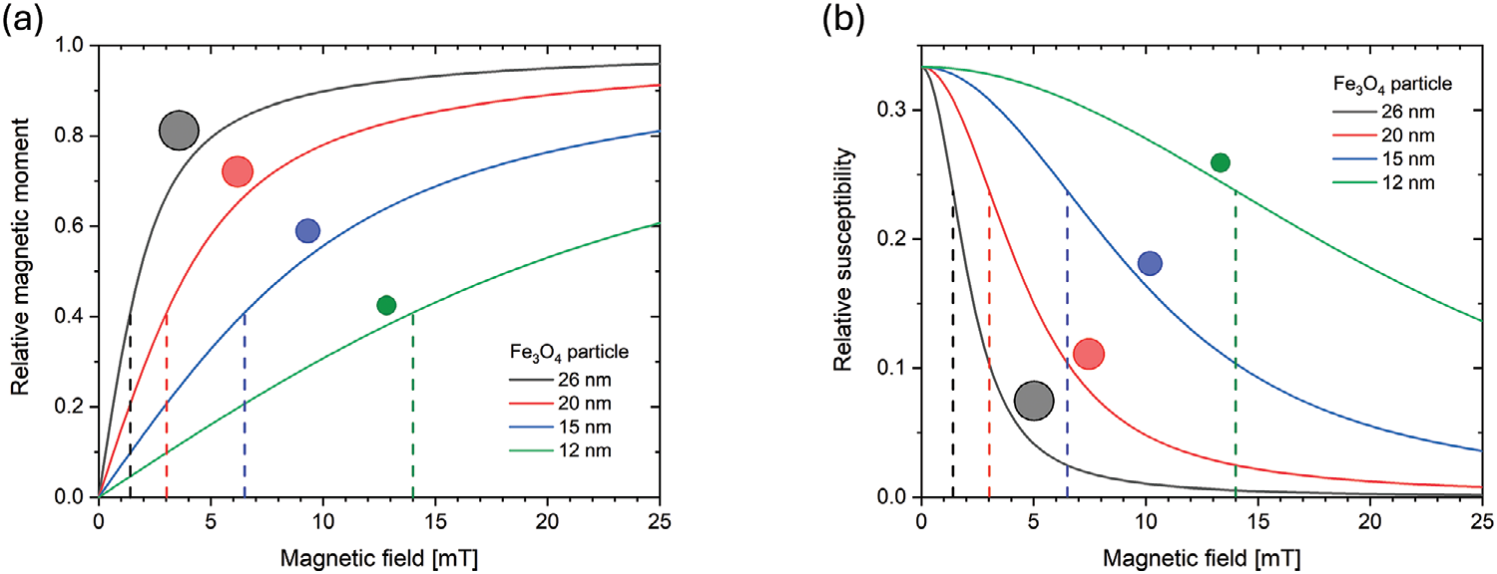
Field‐dependent magnetic properties of spherical magnetite/Fe_3_O_4_ nanoparticles as a function of the magnetic field, for different particle sizes, at 45 °C temperature (typical temperature inside a measurement head). The saturation magnetic moment corresponding to the particle size is given in the legend. (a) Average relative magnetic moment and (b) particle susceptibility. The dashed lines at *ξ* = 1.37 (this value is taken from Table 1 in Section 3.1.1) indicate (a) the field at which the curvature is at its maximum and thus denoting the crossover from linear increase to the onset of saturation and (b) the value of steepest susceptibility decline.

Here, L(ξ) denotes the Langevin function (see **Figure** **2**a), given as

(7) $$L\left(\xi \right)=\coth\xi \;-\frac{1}{\xi }\;\;\;\;\overset{\xi \to 0}{\to }\;\;\;\frac{\xi }{3}-\frac{\xi^{3}}{45}+O\left(\xi^{5}\right),$$

*m_p_* (in Am^2^) is the magnetic moment of a single magnetic particle, *k_B_* = 1.38065 × 10^−23^ J/K is Boltzmann's constant, *T* the temperature (in Kelvin), and *M_s_* the saturation magnetization of the particles (in A/m). Saturation magnetization (i.e., full alignment of all magnetic moments with the external field) is achieved either at very high fields or at very low temperatures. The argument of the Langevin function, L(ξ), the dimensionless field parameter ξ can be expressed and rewritten using the magnetic particle's individual moment (Equation 4) as follows:

(8) $$\xi =\mu_{0}\;\frac{m_{p}}{k_{B}T}\cdot H\;=\mu_{0}\;\frac{M_{s}\;\pi \;d_{c}^{3}}{6k_{B}T}\cdot H$$

and either be described by a (scaled) magnetic field, or equivalently by a particle moment. The Langevin magnetization curve, *M*(*H*), is nonlinear, non‐hysteretic, and approaches saturation magnetization, *M_S_*, for large positive and negative fields, and does so faster for larger particle sizes as depicted in Figure 2a.

Note that the Langevin function, Equation (7), is also valid for classical paramagnets, but in that case, the particle moment *m_p_* is replaced by the individual atomic moment μ. As such, MNP is characterized by strong magnetic interaction from atom to atom within the particle, but negligible interaction from particle to particle within the ensemble (s. section above), and the assumptions of thermal equilibrium can be made for small fields and small particles (e.g., |*H*| ≤ 15 mT/µ_0_ for Fe_3_O_4_‐MNP with *d_C_* =  10 nm).Therefore, the Langevin function (7) can then be used for the determination of the MNP's magnetic moment and to compare the average magnetization of the particle core, *M_s_* = *m_p_*/*V_c_*, with the bulk magnetization of the respective material or at the same time to predict the particle core size from a magnetization measurement^[^ ^50^ ^]^ (cf. Figure 2). As we will discuss later throughout this review, the as‐shown size‐dependency of the magnetic response of MNP is of major importance for its application in materials sciences (Section 4.2 on materials characterization for application details).

In literature, the mass magnetization is usually given by dividing *m_p_* by mass of magnetic material. For magnetite (Fe_3_O_4_) particles, a value of *M_s,m_* = 92 Am^2^/kg for the saturation magnetization is given.^[^ ^76^ ^]^ Multiplied with the mass density of magnetite, *ρ* = 5175 kg/m^3^, a volume magnetization of *M_s_* = 476.1 kA/m is obtained. The linear susceptibility of the particles is given by the derivative of the magnetization with respect to magnetic field, as seen from Equation (1b) and plotted in Figure 2b. From the position of the inflection points of steepest decline in χ(*d_C_*) (as marked in Figure 2b) one directly sees that the linear relationship of *M*(*H*) given by Equation (1) holds for even higher fields the smaller the particles that are being probed are.

Note that the description of M(L(ξ(H))) in thermal equilibrium is only truly valid for static applied fields, as otherwise (in reality), the particle moments undergo relaxation processes. Therefore, the next section will describe these processes dynamically under non‐equilibrium conditions as a response of MNP to a time‐varying external field *H*(*t*).

#### 2.1.2 Dynamic Magnetization: Description Under Non‐Equilibrium Conditions with Particle Relaxation Physics

MNP exposed to an external sinusoidal magnetic field

(9) $$H_{ac}\;\left(t\right)=H_{0}\;\cdot \cos\left(2\pi f\cdot t\right)$$

with the field amplitude, *H*_0_, the frequency, *f*, and the time of exposure, *t*, are beginning to rotate with the applied AFM. Simultaneously, their internal magnetic moment ***m***_***p***_ aligns with the AFM. The higher the thermal energy ε_therm_ = *k*_B_ *T*, the less this alignment takes place, i.e., the more the magentic alignment is reduced by competing with thermal activation. When considering the superposition of these two independent alignment processes, i.e., the physical rotation and the intrinsic magnetic moment alignment of each particle with the direction of the AFM, is denoted as relaxation. This means that there are two ways for the magnetic moment of a particle to relax, i.e., follow the AMF: *Brownian relaxation*, which describes the rotation of the particle with respect to its external environment, and *Néel relaxation*, describing the internal alignment of the magnetic moment.^[^ ^27^, ^55^ ^]^ At zero‐field, Brownian and Néel relaxation can be described by their characteristic relaxation times, τ_B_ and τ_N_, respectively, given by:^[^ ^1^ ^]^

(10) $$\tau_{B}=\frac{\pi \eta d_{H}^{3}}{2k_{B}T}\;$$

(11) $$\tau_{N}=\tau_{0}\;\cdot \;\exp\left(\frac{\Delta E}{k_{B}T}\right)$$

With a relaxation time constant τ_0_, the anisotropy energy barrier Δ*E*  = *K*_eff_  · *V*_C_, the viscosity of the carrier liquid η, and the particle hydrodynamic size *d*_H_. As noted above, the saturation magnetization of MNP can be described in terms of the Langevin function (Equation 5 and 6). However, *L*(ξ) does not account for magnetic anisotropy, which describes preferential directions of alignment of the magnetic moments within MNP.^[^ ^57^ ^]^ Furthermore, it is valid only at thermal equilibrium and does not consider particle relaxation processes (see section 2.1 above). Only if the change in the magnetic applied field is slow enough for the magnetization of the MNP to follow the field, i.e., the relaxation time τ is much smaller than applied field frequency *f*, $\tau \ll \frac{1}{f}$, can relaxation effects be neglected and *L*(ξ) accurately describes the magnetization of the MNP.^[^ ^75^ ^]^ In this case, the saturation magnetization of MNP is described by the equilibrium approach given above. However, under non‐equilibrium conditions, where $\tau \;∼\frac{1}{f}$ holds, particle relaxation is driven by the applied field ***H*** as well as influenced by magnetic anisotropy and additionally by thermal fluctuations, overall resulting in hysteresis effects (i.e., an opening of the hysteresis loop) as a direct result of considering the relaxation mechanisms. These relaxation contributions to the MNP saturation magnetization are accurately modeled only by combined Néel‐Brownian rotation relaxation dynamics, which are described mathematically in the following: Consider a single particle, i, with the intrinsic magnetic moment, ***m**_p_*. This magnetic moments’ internal response (i.e., Néel relaxation) to an applied magnetic field, ***H***_eff_, is described by the Landau–Lifshitz–Gilbert (LLG) equation:^[^ ^77^ ^]^

(12) $$\frac{dm_{p,i}}{dt}=\frac{\gamma_{0}}{1+\alpha^{2}}\;\cdot \left(H_{\mathrm{eff}}\times m_{p}+\alpha \cdot m_{p}\times \left(H_{\mathrm{eff}}\times m_{p}\right)\right)$$

with the electron gyromagnetic ratio γ_0_, the (phenomenological) damping parameter α ∈ [0,  1] and the effective field ***H***_eff_ (defined below, Equation 13). The Brownian external rotation dynamics are described by a generalized torque, ***Θ***, acting on the easy axis of a particle, ***n***, and furthermore dependent on the surrounding matter viscosity, η, as well as the hydrodynamic volume, $V_{H}=\frac{\pi }{6}\;\cdot d_{H}^{3}$:^[^ ^78^ ^]^

(13) $$\frac{dn_{i}}{dt}=\frac{\Theta }{6\eta V_{H}}\;\times n$$

Combining these two differential equations (Equations 12 and 13), fully describes the Néel‐Brownian rotation relaxation dynamics for the general case of non‐zero fields and at non‐equilibrium conditions. The physics governing the relaxation process is encoded in ***H***_eff_ and ***Θ***, which are determined using the Helmholtz free energy *F*  =  *U*  − *T* · Σ of the system, with the internal energy *U*, temperature *T* and entropy Σ. Assuming monodisperse MNP, entropy is negligible, so that Σ ≈ 0, and the effective field and generalized torque read:^[^ ^79^, ^80^ ^]^

(14) $$H_{\mathrm{eff}}=\frac{1}{\mu_{0}}\;\cdot \frac{\partial F}{\partial m_{p}}\approx \frac{1}{\mu_{0}}\cdot \frac{\partial U}{\partial m_{p}}$$

and

(15) $$\Theta \;=\frac{\partial F}{\partial n}\;\times n\approx \frac{\partial U}{\partial n}\times n.$$

### 2.2 Principles of Frequency Mixing Magnetic Detection (FMMD)

In FMMD, superparamagnetic MNP are magnetized to or close to saturation by a time‐varying magnetic field consisting of two spectral components: A high frequency (HF) excitation field of frequency *f*_1_ and amplitude *B*_1_ = *µ*_0_ *H*_1_ and a low frequency (LF) drive field with *f*_2_ < *f*_1_ and *B*_2_  =  μ_0_ *H*_2_ > *B*_1_ are simultaneously incident on the sample containing MNP. This causes nonlinear magnetization curves of the particles from which the intermodulation products can be measured, which allow the quantification and interpretation of MNP across a large dynamic measurement range. By applying a static magnetic offset field, denoted by *B*_0_ = *µ*_0_ *H*_0,_ to the sample, the intermodulation can be further fine‐tuned, allowing highly differentiable measurements with high sensitivity. The total time‐varying, dual frequency magnetic field at the sample is then given by superposition as

(16) $$\mu_{0}H\;\left(t\right)=B_{0}+B_{1}\sin\left(2\pi f_{1}\cdot t\right)+B_{2}\sin\left(2\pi f_{2}\cdot t\right).$$

**Figure 3:**
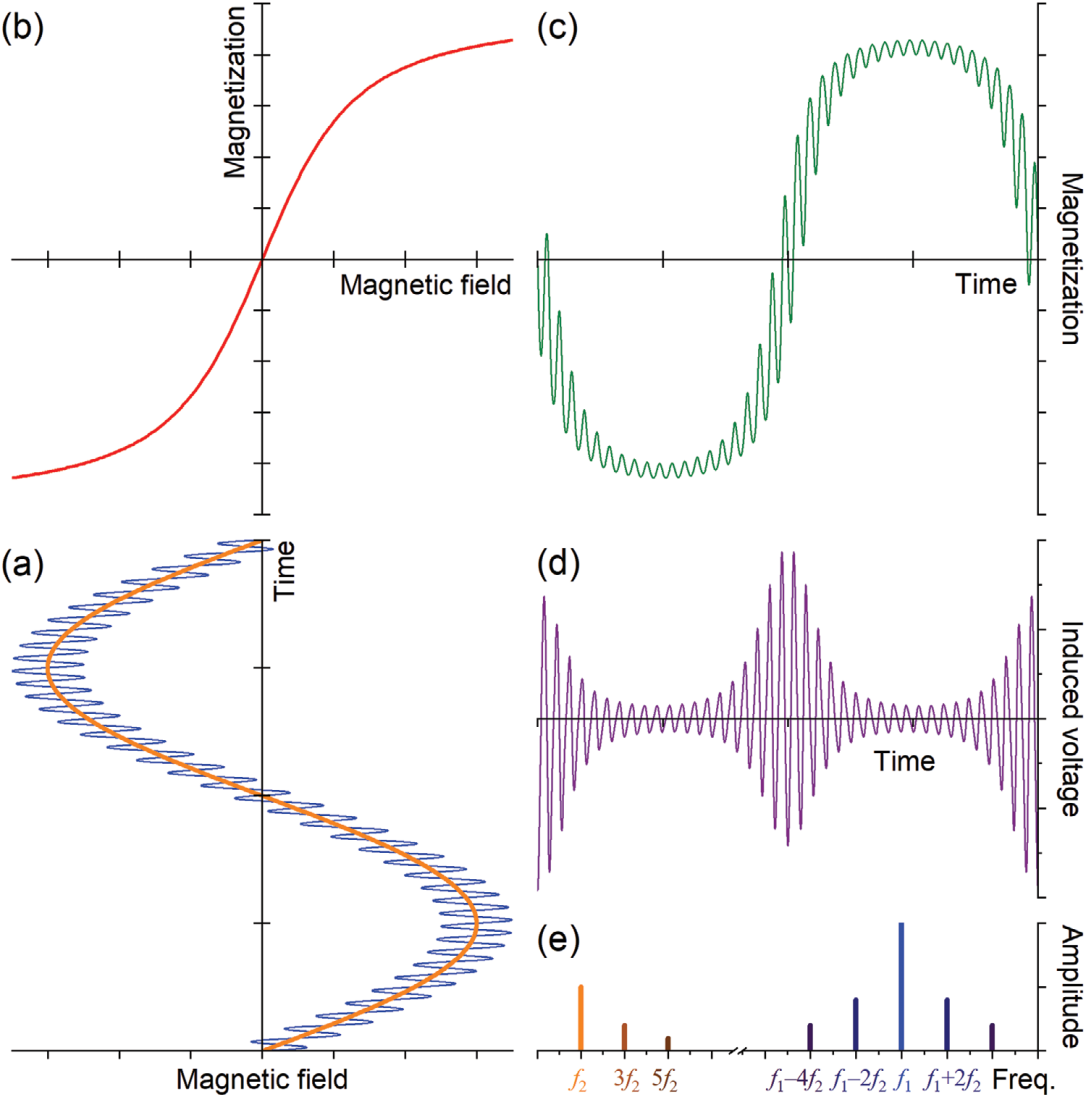
The magnetic response of MNP in FMMD: (a) When a dual‐frequency magnetic field consisting of a high frequency component *f*_1_ and a low frequency component *f*_2_ is applied to superparamagnetic MNP, their nonlinear magnetization (b) leads to a distorted (“flattened”) magnetization response (c) which saturates at high(er) field amplitudes. The induced voltage (d) in the detection coil is given by the time derivative of the particles’ magnetization. In the frequency spectrum (e) of the induced voltage, not only the excitation frequency lines *f*_2_ and *f*_1_ are observed, but also their higher harmonics (e.g., 3*f*_2_, 5*f*_2_, 3*f*_1_, …) and frequency mixing components (e.g., *f*_1_–2*f*_2_, *f*_1_+2*f*_2_, …) are found in the Fourier‐transformed response signal.

The effect of such an applied field on an ensemble of MNP is developed in **Figure** **3** and explains step‐by‐step the appearance of so‐called frequency mixing components as a direct measure to characterize the intermodulation products in FMMD: Let us assume for simplicity a time‐varying excitation field with zero offset field, i.e., *B*_0_ = 0, as given in blue in Figure 3a. Furthermore, assuming that the drive field amplitude is able to drive the MNP (at least toward) to saturation (i.e., *B*_2_ is larger than the field at the dashed lines in Figure 2a for the respective MNP size), then the superparamagnetic MNP magnetizes nonlinearly as shown in Figure 3b according to the Langevin function, cf. Equation (5). This nonlinear response of the MNP to the applied field directly leads to their distorted time‐varying magnetization according to the blue curve in Figure 3c, which exhibits flattened plateaus (global extrema) with strong gradients (slope in Figure 3c) at the zero‐crossings and overlayed minor variations (local extrema) imposed by the excitation field, *f*_1_,*B*_1_. The Fourier transform of this distorted magnetization response is schematically depicted in Figure 3d, which interestingly does not only exhibit peak‐lines at the excitation components *f*_1_ and *f*_2_ but additionally also at the uneven harmonics *f*_1_, 3*·f*_1_, 5*·f*_1_ …, and *f*_2_, 3*·f*_2_, 5*·f*_2_, as well as furthermore for the mixing terms *f*_1_ ± 2*·f*_2_, *f*_1_ ± 4*·f*_2_, …. The even harmonics 2*·f*_2_, 4*·f*_2_ … and uneven mixing terms such as *f*_1_ ± *f*_2_, *f*_1_ ± 3*·f*_2_ … are forbidden by symmetry because the magnetization curve is point‐symmetric and thus has uneven powers of *H* only in its Taylor expansion about the origin (s. next section for further details). In other words, the superparamagnetic MNP yield responses at mixing frequencies *f*_1_ ± *n*·*f*_2_ (where *n* denotes an even integer number), due to their nonlinear and non‐hysteretic (in the limit of small frequencies below *f* ≈ 10 kHz for MNP with *d_C_* =  10 nm) magnetization.

If, however, a non‐zero static magnetic offset field, *B*_0_, is additionally applied to the sample, the symmetry is broken and both even harmonics 2·*f*_2_, 4·*f*_2_ … and uneven mixing terms such as *f*_1_ ± *f*_2_, *f*_1_ ± 3·*f*_2_ … appear in the response. Their expression depends on the strength of the applied offset field. Note that some mixing components even vanish again for some distinct values of *B*_0_ and in dependence on the MNP intrinsic properties, a feature that can be used for MNP characterization and analysis, outlined in further detail in Section 4.2.

The signal generation is generally identical to that of the more commonly known MNP imaging modality of magnetic particle imaging (MPI), where also the odd harmonics are measured and analyzed but only for single frequency excitation (either for direct quantification or indirect characterization of MNP localized in the MPI scanner).^[^ ^81^, ^82^ ^]^ The intermodulation products from dual frequency excitation available in FMMD are a direct result of a Taylor expansion of the nonlinear magnetization, (as will be shown in Section 3.1 below) and allows for more sensitive detection of MNP signals, i.e., a lower detection limit, than MPI (for details in application see Section 4.1).

The high sensitivity of FMMD originates directly from the afore mentioned nonlinear magnetic moments in FMMD: in fact, the voltage (signal) picked up by the FMMD reader is generally proportional to the negative change of magnetization with the applied field (according to Faraday's law), i.e., the FMMD reader signal intensity yields:^[^ ^83^ ^]^

(17) $$I\left(t\right)\propto V\left(t\right)\propto -M^{\prime }\;\left(t\right)=-\frac{dM\left(t\right)}{dB\left(t\right)}\approx \frac{\Delta M}{\Delta B}$$

**Figure 4:**
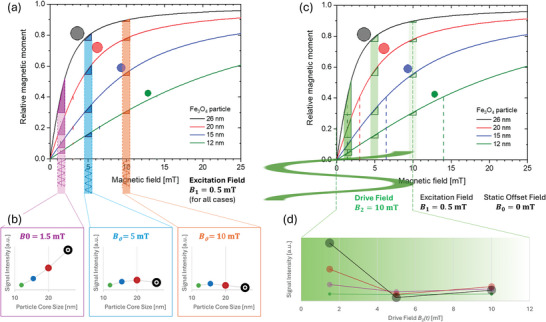
Principle of field‐and‐core‐size dependent magnetic response of MNP to dual frequency excitation. (a) Superparamagnetic MNP magnetization as a function of the applied (single frequency) magnetic field for differently sized magnetite MNP for core sizes ranging *d_C_* =  12 nm to 26 nm (cf. Figure 2). Below, the excitation field with *B*_1_ =  0.5 mT for varying offset fields, *B*_0_, are shown. The dark‐colored triangles approximate the signal intensity for the given excitation field at different static offset field values according to Equation (17). These mark the size‐dependent “corridor of influence” that a specific AMF has on monodisperse MNP. For simplicity, the drive field *B*_2_ is neglected here. (b) FMMD signal generated from approximating the size‐dependent magnetic response capability of the MNP, i.e., change of magnetization per field, $\frac{\Delta M}{\Delta B}\;\left(B_{0}\right)$, shows strong dependence on MNP core size and the specific offset‐field, i.e., the nonlinear section of the magnetization curve probed (based on the triangles shown in panel (a)). (c) Size‐dependent MNP magnetization response for dual‐frequency excitation: The drive field (shown with amplitude *B*_2_ =  10 mT) pushes the “corridor of influence” for the excitation field (again: *B*_1_ =  0.5 mT) along the size‐dependent individual particle magnetization curve in the same way as a static offset field (for the same exciation field), cf. subfigure (a). The triangles of $\frac{\Delta M}{\Delta B}\left(t\right)$ are equivalent to (a), if the same reference fields with *B*_0_ = *B*_2_ (*t*) are chosen. Dynamics are indicated by lighter color, i.e., lighter color represents a field later in time. (d) Approximated size‐dependent FMMD signal for the three exemplary points in time/field amplitudes *B*_2_(*t*) – analogous to (b).

This makes the FMMD signal highly sensitive to any change in the MNP magnetization in dependence on the applied AMF, i.e., to the nonlinear slope of the M‐of‐B curve. The principle is outlined for field and size dependent effects in **Figure** **4**.

Figure 4 intuitively illustrates that depending on the value of field amplitudes chosen, a different part of the magnetization curve of MNP is driven to excitation, i.e., probed: First one concludes that the overall section of the magnetization curve being probed by FMMD is selected, i.e., limited, by the drive field, *B*_2_. This selection can second be shifted by applying the static offset field, as exemplarily shown for three static offset fields in Figure 4a (color shading).

As given by Equation (17), the (negative) amount of change in magnetization per field, Δ*M*/Δ*B*, has direct implication on the signal generation. Analyzing large particles with *d_C_* =  26 nm for example, Figure 4c demonstrates that these particles generate a strong signal at low drive field value, e.g. *B*_2_(*t*)  =  1.5 mT, which diminishes strongly for higher values of *B*_2_(*t*). In general, the signal generated by small particles, e.g. *d_C_* =  12 nm, shows a low signal intensity, independent of the static offset field, i.e., the section of the magnetization curve probed. This is because larger particles reach higher absolute saturation magnetization faster (i.e., show a steeper slope) than small particles (cf. Figure 2, dashed lines).

These observations allow identifying a size‐dependent “sweet‐spot” for optimal FMMD signal generation in dependence of the MNP (mean) core size and size distribution (discussed in more detail in section 3.3.1). Once driven into full saturation (e.g., at approximately *B*  =  25 mT for all but the smallest particle size, *d_C_* =  12 nm in Figure 4a), the particles’ magnetic moment will not generate a FMMD signal anymore as their slope in M‐versus‐H is (almost) zero. As a real MNP sample is always polydisperse, the FMMD signal naturally consists of overlaying contributions for differently sized particles, weighted by the particle size distribution. By selecting size‐optimized combinations of field parameters (*B*_0_, *B*_1_, *B*_2_ and *f*_1_, *f*_2_) applied to MNP samples, their particles sizes can be backwardly derived from decomposing their individual magnetization response from the FMMD signal according to the principle given above. Its applications in biomedicine and materials characterization are discussed in detail in chapter 4.

In summary, Figure 4 demonstrates that the response of an ensemble of MNP under investigation is governed by its constituent core sizes (determining the saturation magnetization and steepness of slope to reach saturation) but the selection of this response accessible to FMMD, i.e., the section fo the magnetization curve being probed, is dependent on the field parameters chosen.

**Figure 5:**
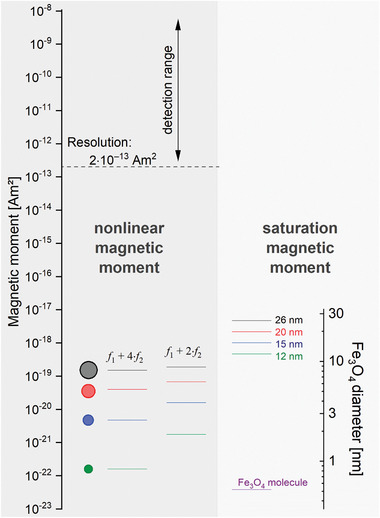
Illustration of the magnetic moment detection scales for individual particles of magnetite/Fe_3_O_4_ ranging from 1 *µ_B_* (= 0.5 × 10^−22^ Am^2^) at the bottom to 10^15^ *µ_B_* (= 10^−8^ Am^2^) at the top. (dark gray) The saturation moments of a single Fe_3_O_4_ molecule and of particles with exemplary sizes between 12 nm and 26 nm are represented by the colored horizontal lines. (light gray) The maximum achievable nonlinear magnetic moments of these particles at the mixing components *f*_1_ + 2·*f*_2_ and *f*_1_ + 4·*f*_2_ at typical FMMD excitation fields are shown by the horizontal lines at the left, together with a proportional visualization of their sizes. Note that the nonlinear moments are 30‐ to 3000‐fold smaller than the saturation moments. The theoretical magnetic moment resolution of an FMMD device is indicated by the dashed line in black.

The saturation magnetic moments are several orders of magnitude larger compared to the intermodulation magnetic moments of an FMMD measurement. This is comprehensively illustrated in **Figure** **5** for Fe_3_O_4_‐MNP ranging from a single magnetite molecule to a MNP sized between $d_{C}=\left(12,\text{…},\;26\;\mathrm{nm}\right)$ (approaching the limit of superparamagnetism) showing that the frequency‐mixing components *f*_1_ + 2*f*_2_ and *f*_1_ + 4*f*_2_ are well below the saturation magnetic moments and the detection range extends over more than four orders of magnitude. Even more so, the nonlinear intermodulation moments decrease with increasing order of the frequency mixing components, e.g., the components *f*_1_ + 4·*f*_2_ are smaller than the *f*_1_ + 2·*f*_2_ components (see Figure 5). The decrease is much stronger for the nonlinear magnetic intermodulation moments, further reflecting the great potential for highly sensitive detection of small MNP with dual‐frequency excitation.

Particularly for very small particles with a diameter below 10 nm, the nonlinear magnetic moments decrease very rapidly with decreasing core diameters. For such small particles, the nonlinear moments at *f*_1_ + *f*_2_ and *f*_1_ + 2*f*_2_ scale with the 12^th^ power of the diameter, see Supporting Information, section C. The nonlinear moments at *f*_1_ + 3*f*_2_ and *f*_1_ + 4*f*_2_ even scale with the diameter to power 18.

Note that both the saturation magnetization and the nonlinear magnetic moments decrease for smaller sizes, as a direct consequence of Equation (5), in accordance with the size‐and‐field‐dependent behavior of MNP already discussed above (cf. Figure 4).

The theoretical detection limit of a common FMMD reader (9 mm diameter pickup coil) is given within the Nyquist noise limit with approximately 2 × 10^−13^ Am^2^, calculated from first physical principles,^[^ ^42^ ^]^ which is up to two orders of magnitude below the detection limit of a common SQUID magnetometer applied to MNP.^[^ ^60^, ^84^ ^]^ From Figure 5, one directly sees that several millions of particles are required to obtain a measurable signal in the detection range of FMMD, i.e., approximately one million of the 26 nm sized particles, or about 100 million of the 12 nm particles are needed to reach the detection limit. This is reasonable from the implications just discussed above: since larger sized particles show a steeper increase in the M‐of‐B‐curve (cf. Figure 4), they consequently display a stronger signal *V*(*t*)∝*M*′ (*t*) =  Δ*M*/Δ*B* and therefore less larger particles are needed to generate the same signal intensity as a larger amount of small particles.

## 3 Modeling the FMMD Signal

In the following, we present state‐of‐the‐art methods to mathematically describe (part of) the FMMD signal generation. Therefore, we differentiate between single size (monodisperse) MNP (see section 3.1) and size‐distributed MNP (see section 3.2). We start most intuitively by presenting the expansion of the Langevin function for small excitation fields in equilibrium conditions assuming a linear response (Section 3.1.1). Despite being quite far from describing the reality of MNP excitation, the Taylor expansion allows a fundamental understanding of the field dependency of the FMMD signal. Next, we explore the nonlinear response of MNP in equilibrium for arbitrary amplitudes (Sections 3.1.2 and 3.2.1 for polydisperse MNP), also dealing with the special case of small, almost zero field amplitudes. From these, we will furthermore derive characteristics to evaluate experimental FMMD signals. Finally, implementing more complete but complex particle dynamic relaxation simulations is discussed in Section 3.1.3 (Section 3.2.2 for polydisperse MNP), presenting an elaborated way of predicting nonlinear MNP response under non‐equilibrium conditions with a Monte Carlo method. Where applicable, we also verify our modeling approaches and discuss their validity along the way. The chapter concludes with a brief tabular comparison of capabilities, limitations, and application examples of the three above‐mentioned models for FMMD signal generation in Section 3.3.

### 3.1 Fundamental Approaches for Monodisperse MNP

#### 3.1.1 Linear Response under Equilibrium Conditions: Taylor Approximation for Small Excitation Fields

In the following, we formulate a mathematical description of the linear magnetic response of MNP in thermal equilibrium to a dual frequency excitation using the superparamagnetic behavior as described by the Langevin function (Equation 6). In the limit of small excitation field amplitudes and/or small particles, ξ∼0 holds for the argument of the Langevin function (see Equation 8) and the function can be Taylor expanded:

(18) $$\begin{array}{cc} L\;\left(\xi \right) & =L\left(\xi_{0}\right)+{\left.\frac{dL}{d\xi }\right|}_{\xi =\xi_{0}\;}\cdot \left(\xi -\xi_{0}\right)+{\left.\frac{1}{2}\frac{d^{2}L}{d\xi^{2}}\right|}_{\xi =\xi_{0}\;}\\ & \;\cdot {\left(\xi -\xi_{0}\right)}^{2}+{\left.\frac{1}{6}\frac{d^{3}L}{d\xi^{3}}\right|}_{\xi =\xi_{0}\;}\cdot {\left(\xi -\xi_{0}\right)}^{3}+{\left.\frac{1}{24}\frac{d^{4}L}{d\xi^{4}}\right|}_{\xi =\xi_{0}\;}\\ & \;\cdot {\left(\xi -\xi_{0}\right)}^{4}+{\left.\frac{1}{120}\frac{d^{5}L}{d\xi^{5}}\right|}_{\xi =\xi_{0}\;}\cdot {\left(\xi -\xi_{0}\right)}^{5}+\cdot \cdot \cdot \end{array}$$

with the derivatives as follows:

(19) $$\frac{dL\left(\xi \right)}{d\xi }=\frac{1}{\xi^{2}}\;-\frac{1}{\sinh^{2}\xi }\;\;\;\;\overset{\xi \to 0}{\to }\;\;\;\frac{1}{3}-\frac{\xi^{2}}{15}+\frac{2\xi^{4}}{189}-O\left(\xi^{6}\right)$$

(20) $$\frac{d^{2}L\left(\xi \right)}{d\xi^{2}}=\frac{2\coth\;\xi }{\sinh^{2}\xi }\;-\frac{2}{\xi^{3}}\;\;\;\overset{\xi \to 0}{\to }\;\;\;-\frac{2\xi }{15}+\frac{8\xi^{3}}{189}-O\left(\xi^{5}\right)$$

(21) $$\begin{array}{cc} \frac{d^{3}L\left(\xi \right)}{dx^{3}} & =\frac{6}{\xi^{4}}\;-\frac{4\coth^{2}\xi }{\sinh^{2}\xi }-\frac{2}{\sinh^{4}\xi }\;\;\;\overset{\xi \to 0}{\to }\\ & \;-\frac{2}{15}+\frac{8\xi^{2}}{63}-\frac{2\xi^{4}}{45}+O\left(\xi^{6}\right) \end{array}$$

(22) $$\begin{array}{cc} \frac{d^{4}L\left(\xi \right)}{d\xi^{4}} & =\frac{16\coth\;\xi }{\sinh^{4}\xi }+\frac{8\coth^{3}\;\xi }{\sinh^{2}\xi }-\frac{24}{\xi^{5}}\;\;\;\overset{\xi \to 0}{\to }\\ & \;\frac{16\xi }{63}-\frac{8\xi^{3}}{45}+O\left(\xi^{5}\right) \end{array}$$

(23) $$\begin{array}{cc} \frac{d^{5}L\left(\xi \right)}{dx^{5}} & =\frac{120}{\xi^{6}}\;-\frac{16\coth^{4}\xi }{\sinh^{2}\xi }-\frac{88\coth^{2}\xi }{\sinh^{4}\xi }-\frac{16}{\sinh^{6}\xi }\;\;\;\overset{\xi \to 0}{\to }\\ & \;\frac{16}{63}-\frac{8\xi^{2}}{15}+\frac{32\xi^{4}}{99}-O\left(\xi^{6}\right) \end{array}$$

The magnetic particles are exposed to a magnetic field consisting of two distinct excitation frequencies *f*_1_ and *f*_2_ (with *f*_1_ > *f*_2_) with amplitudes *B*_1_ and *B*_2_ (with *B*_1_ < *B*_2_), and a static magnetic offset field *B*_0_, as given in Equation (16). Thus, when multiplied out, it becomes obvious that the quadratic term, (ξ − ξ_0_)^2^, in the Taylor expansion contains the sum frequency term *f*_1_ + *f*_2_ (among others):

(24) $$\begin{array}{cc} & {\left[B_{1}\sin\left(2\pi f_{1}t\right)+B_{2}\sin\left(2\pi f_{2}t\right)\right]}^{2}\\ & \;=\cdot \cdot \cdot -B_{1}B_{2}\cos\left[2\pi \left(f_{1}+f_{2}\right)\;t\right]+\cdot \cdot \cdot \end{array}$$

Analogously, the cubic term, (ξ − ξ_0_)^3^, yields the component *f*_1_ + 2·*f*_2_, the quartic term, (ξ − ξ_0_)^4^, term leads to the component *f*_1_ + 3·*f*_2_, and finally the quintic term, (ξ − ξ_0_)^5^, evolves the component *f*_1_ + 4·*f*_2_, as follows:

(25) $$\begin{array}{cc} & {\left[B_{1}\sin\left(2\pi f_{1}t\right)+B_{2}\sin\left(2\pi f_{2}t\right)\right]}^{3}\\ & \;=\cdot \cdot \cdot -\frac{3}{4}B_{1}B_{2}^{2}\sin\left[2\pi \left(f_{1}+2\cdot f_{2}\right)\;t\right]+\cdot \cdot \cdot \end{array}$$

(26) $$\begin{array}{cc} & {\left[B_{1}\sin\left(2\pi f_{1}t\right)+B_{2}\sin\left(2\pi f_{2}t\right)\right]}^{4}\\ & \;=\cdot \cdot \cdot +\frac{1}{2}B_{1}B_{2}^{3}\cos\left[2\pi \left(f_{1}+3\cdot f_{2}\right)\;t\right]+\cdot \cdot \cdot \end{array}$$

(27) $$\begin{array}{cc} & {\left[B_{1}\sin\left(2\pi f_{1}t\right)+B_{2}\sin\left(2\pi f_{2}t\right)\right]}^{5}\\ & \;=\cdot \cdot \cdot +\frac{5}{16}B_{1}B_{2}^{4}\sin\left[2\pi \left(f_{1}+4\cdot f_{2}\right)\;t\right]+\cdot \cdot \cdot \end{array}$$

Note that the second order mixing component *f*_1_ + *f*_2_ and the fourth‐order component *f*_1_ + 3·*f*_2_ may only appear if there is a non‐vanishing static offset field *B*_0,_ due to symmetry. Mathematically, this is because at zero offset field with *B*_0_ = 0 (and thus expansion point *ξ*_0_ = 0) the corresponding even Langevin derivatives $L^{\prime \prime }$ and L ^(4)^ are also zero. When considering these four mixing components (Equations (24), (25), (26), (27)) individually, one can derive characteristic features from the shape of each component's L(ξ)‐curve. In first approximation, minima, maxima and zero‐crossings are chosen as characteristic features of each mixing component in dependence of the dimensionless parameter ξ, as is comprehensively summarized in the **Table** **1**. As seen there, higher order mixing terms display more and more characteristic features for individual identification and information readout. This demonstrates the highly differentiable and multi‐faceted informational content that a FMMD signal offers for the characterization of MNP and furthermore in selectivity for application.

**Table 1:** Individual derivatives of the Langevin function representing the first harmonic (*f*_1_) as well as the first through fourth intermodulation mixing terms (*f*_1_ + *nf*_2_, *n*  =  1, 2, 3, 4) for small excitation amplitudes. Their functional dependency is plotted as derivatives of the Langevin function. The values *ξ* of the first five characteristic features (maxima, minima, and zero crossings) are listed and depicted (and numbered) as blue points in the plots for comparison.

Mixing term	Feat. #1	Feat. #2	Feat. #3	Feat. #4	Feat. #5	Functional dependence
1						
*f*_1_ *ξ* L ′(*ξ*)	Max. 0 0.33333	n/a	n/a	n/a	n/a	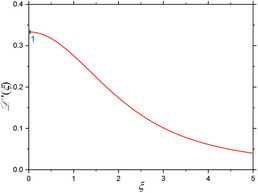
2						
*f*_1_ ± *f*_2_ *ξ* L ′′(*ξ*)	Zero 0 0	Min. 1.37225 −0.10596	n/a	n/a	n/a	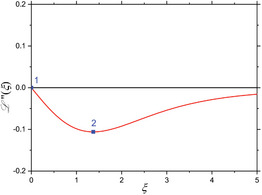
3						
*f*_1_ ± 2·*f*_2_ *ξ* L ′′′(*ξ*)	Min. 0 −0.13333	Zero 1.37225 0	Max. 2.36074 0.04029	– n/a	n/a	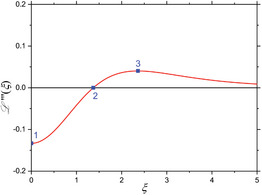
4						
*f*_1_ ± 3·*f*_2_ *ξ* L ^(4)^(*ξ*)	Zero 0 0	Max. 0.84991 0.13065	Zero 2.36074 0	Min. 3.21188 −0.01693	n/a	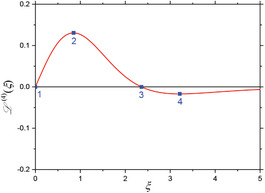
5						
*f*_1_ ± 4·*f*_2_ *ξ* L ^(5)^(*ξ*)	Max. 0 0.25397	Zero 0.84991 0	Min. 1.52078 −0.11957	Zero 3.21188 0	Max. 3.99369 0.00760	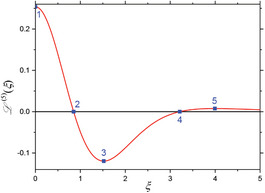

Note that when the static magnetic offset field *B*_0_, corresponding to the dimensionless variable *ξ*_0_, is changed, the signal amplitudes and phases of the frequency mixing terms change according to the corresponding derivative, as depicted in Table 1. Especially the 180° phase reversals at the zero crossings are experimentally well detectable and therefore a useful indication feature for MNP characterization (see chapter 4 for a detailed application).

#### 3.1.2 Nonlinear Response under Equilibrium Conditions: Modeling the Magnetic Particle Moment

##### Small Near‐Zero Excitation Amplitudes: Using Langevin Function Derivatives

Consider a single MNP with saturation magnetic moment *m_p_* that is exposed to a two‐frequency excitation field with offset according to Equation (16). If both the high frequency amplitude *B*_1_ and the low frequency amplitude *B*_2_ are chosen sufficiently small (i.e., well below 1 mT), the Taylor expansion of the Langevin function, Equation (18), is still valid, from which the following expression is obtained for the particle's time‐dependent magnetic moment by inserting the powers of Equation (16):

(28) $$\begin{array}{c} \begin{array}{cc} \frac{m\left(t\right)}{m_{p}} & =L\left(\frac{m_{p}B_{0}}{k_{B}T}\right)+L^{\prime }\left(\frac{m_{p}B_{0}}{k_{B}T}\right)\cdot \frac{m_{p}}{k_{B}T}\cdot B_{1}\cdot \sin\left(2\pi f_{1}t\right)\\ & \;-\frac{1}{2}L^{\prime \prime }\left(\frac{m_{p}B_{0}}{k_{B}T}\right)\cdot {\left(\frac{m_{p}}{k_{B}T}\right)}^{2}\cdot B_{1}B_{2}\cdot \cos\left(2\pi \left(f_{1}+f_{2}\right)t\right)\\ & \;-\frac{1}{8}L^{\prime \prime \prime }\left(\frac{m_{p}B_{0}}{k_{B}T}\right)\cdot {\left(\frac{m_{p}}{k_{B}T}\right)}^{3}\cdot B_{1}B_{2}^{2}\cdot \sin\left(2\pi \left(f_{1}+2f_{2}\right)t\right)\\ & \;+\frac{1}{48}L^{\left(4\right)}\left(\frac{m_{p}B_{0}}{k_{B}T}\right)\cdot {\left(\frac{m_{p}}{k_{B}T}\right)}^{4}\cdot B_{1}B_{2}^{3}\cdot \cos\left(2\pi \left(f_{1}+3f_{2}\right)t\right)\\ & \;+\frac{1}{384}L^{\left(5\right)}\left(\frac{m_{p}B_{0}}{k_{B}T}\right)\cdot {\left(\frac{m_{p}}{k_{B}T}\right)}^{5}\cdot B_{1}B_{2}^{4}\cdot \sin\left(2\pi \left(f_{1}+4f_{2}\right)t\right)+\cdot \cdot \cdot \end{array} \end{array}$$

The prefactor of the *n* ^th^‐order Taylor expansion term is $\frac{1}{n\;!}$ and the prefactor of the (ξ − ξ_0_)^*n*^ term is $\frac{n}{2^{n-1}}$, yielding a combined prefactor of $\frac{1}{\left(n-1\right)\;!\;2^{n-1}}$ for the *n* ^th^‐order term that corresponds to the mixing terms *f*_1_ ± (*n* − 1)*f*_2_. Here, only the positive mixing terms at frequencies *f*_1_ + *n*·*f*_2_ are listed, the also prevalent negative mixing terms *f*_1_ – *n*·*f*_2_ are omitted for the sake of clarity. They have the same scaling factors.

##### Arbitrary (Large) Excitation Amplitudes: Using Demodulated Langevin Functions

Using higher excitation amplitudes, *B_i_* > 1 mT, the Taylor expansion is no longer valid, as also experimentally demonstrated from deviations of the offset field dependence from the Langevin derivatives.^[^ ^74^ ^]^ This limitation can be overcome as follows: The nonlinear magnetic response moment of a particle to a two‐frequency excitation field is calculated using the following expression

(29) $$\begin{array}{cc} m_{NL} & =m_{p}\;\cdot L\left[\frac{m_{p}}{k_{B}T}\left(B_{0}+B_{1}\sin\left(2\pi f_{1}t\right)+B_{2}\sin\left(2\pi f_{2}t\right)\right)\right]\\ & \;\times \cos\left(2\pi \left(f_{1}+f_{2}\right)t_{i}\right). \end{array}$$

The desired mixing component is demodulated by multiplying with the sine or the cosine of the mixing frequency. If *f*_1_ is chosen as an integer multiple of *f*_2_, then also the mixing frequency *f*_1_ + *n* · *f*_2_ is an integer multiple of *f*_2_, as proven in the literature already.^[^ ^85^ ^]^ Therefore, the demodulation can be performed numerically as a summation over a full period of the low frequency *f*_2_, which covers also full periods of the high frequency *f*_1_ and of the mixing frequency *f*_1_ + *n* · *f*_2_. The nonlinear magnetic moment at the first mixing frequency *f*_1_ + *f*_2_ then follows as:

(30) $$\begin{array}{cc} m_{f_{1}+f_{2}} & =m_{p}\;\cdot \frac{2}{n}\sum_{i=0}^{n-1}L\left[\frac{m_{p}}{k_{B}T}\left(B_{0}+B_{1}\sin\left(2\pi f_{1}t_{i}\right)+B_{2}\sin\left(2\pi f_{2}t_{i}\right)\right)\right]\\ & \;\times \cdot \cos\left(2\pi \left(f_{1}+f_{2}\right)t_{i}\right). \end{array}$$

The additional factor of 2/*n* in front of the summation of Equation (28) normalizes the sum and accounts for the fact that the average of sin^2^ or cos^2^ over a full period is ½. The sampling time steps, *t_i_*, should be chosen according to the high frequency period $T_{1}=\frac{1}{f_{1}}$: For example, taking 10 steps in a period 1/*f*_1_, i.e., Δ*t* = *t_i_* − *t_i_*_–1_ = 0.1/ *f*_1_, yielded sufficient numerical precision. Even though in practice, the ratio of *f*_1_ to *f*_2_ is in the range from 500 to 1000, it proved sufficient to perform the calculations with *f*_1_ =  20 · *f*_2_ with much less computational effort, yielding almost identical results. From this assumption, *n* = 200 was applied in our calculations shown here.

The higher order mixing terms are as follows:

(31) $$\begin{array}{cc} m_{f_{1}+2f_{2}} & =m_{p}\;\cdot \frac{2}{n}\sum_{i=0}^{n-1}L\left[\frac{m_{p}}{k_{B}T}\left(B_{0}+B_{1}\sin\left(2\pi f_{1}t_{i}\right)+B_{2}\sin\left(2\pi f_{2}t_{i}\right)\right)\right]\\ & \;\times \cdot \sin\left(2\pi \left(f_{1}+2f_{2}\right)t_{i}\right) \end{array}$$

(32) $$\begin{array}{cc} m_{f_{1}+3f_{2}} & =m_{p}\;\cdot \frac{2}{n}\sum_{i=0}^{n-1}L\left[\frac{m_{p}}{k_{B}T}\left(B_{0}+B_{1}\sin\left(2\pi f_{1}t_{i}\right)+B_{2}\sin\left(2\pi f_{2}t_{i}\right)\right)\right]\\ & \;\times \cdot \cos\left(2\pi \left(f_{1}+3f_{2}\right)t_{i}\right) \end{array}$$

(33) $$\begin{array}{cc} m_{f_{1}+4f_{2}} & =m_{p}\;\cdot \frac{2}{n}\sum_{i=0}^{n-1}L\left[\frac{m_{p}}{k_{B}T}\left(B_{0}+B_{1}\sin\left(2\pi f_{1}t_{i}\right)+B_{2}\sin\left(2\pi f_{2}t_{i}\right)\right)\right]\\ & \;\times \cdot \sin\left(2\pi \left(f_{1}+4f_{2}\right)t_{i}\right) \end{array}$$

Note the alternation between sin(.) and cos(.) as demodulation reference functions between even and odd mixing terms. They are caused by the alternating nonlinear magnetic moments switching from even to uneven functions, as already seen in equation ((28)‐(23)) and Table 1. Agreement between the small amplitude approximation, equation (28), and the numerical calculations of equation (30) to (33) for large amplitudes was verified, see Supporting Information, section B.

#### 3.1.3 Nonlinear Response under Non‐Equilibrium Conditions: Implementing Particle Dynamic Relaxation Simulations

Based on the description of magnetization dynamics driven by particle relaxation from Section 2.1.2, let us consider the internal energy of a single particle, *U*. It includes contributions arising from the applied field ***H*** (ε_Zee_), particle interaction (ε_pp − IA_) and magnetic anisotropy (ε_ai_):^[^ ^59^, ^75^ ^]^

(34) $$U=\varepsilon_{\mathrm{Zee}}+\varepsilon_{\mathrm{pp}-\mathrm{IA}}+\varepsilon_{ai}$$

with the Zeeman term ε_Zee_ =   − ***m*** · ***H***, the magnetic anisotropy energy ε_*ai*_ = K_eff_ *V_M_* · (***m*** · ***n***)^2^ as well as the magnetic interaction energy (ε_pp − IA_) that summarizes the magnetic dipole‐dipole interaction exerted on an single particle by all its surrounding MNP. For a single particle with magnetic moment ***m*_0_** and at a distance ***r**_i_* to all other MNP with a magnetic moment ***m**_i_*, ε_pp − IA_ this interaction reads:

(35) $$\varepsilon_{pp-IA}=\sum_{i}\frac{\mu_{0}}{4\pi r_{i}^{3}}\cdot \left(\frac{3\left(m_{0}\cdot r_{i}\right)\cdot \left(m_{i}\cdot r_{i}\right)}{r_{i}^{2}}-m_{0}\cdot m_{i}\right).\;$$

The inner energy *U* couples Equation (12) through (15), and thus describes the combined Néel and Brownian relaxation dynamics fully. However, in order to include thermal fluctuations, stochastic terms must be introduced to Equations (14) and (15), giving:^[^ ^57^ ^]^

(36) $$H_{eff}=\frac{1}{\mu_{0}}\;\cdot \frac{\partial U}{\partial m}+H_{th}$$

and

(37) $$\Theta \;=\frac{\partial U}{\partial n}\;\times n+\Theta_{th}$$

These thermally generated fields, ***H***_th_, and torques, **Θ**_th_, can be described as white noise (mathematically described as a Gaussian‐distribution) defined under the initial conditions as:

(38) $$H_{th}^{i}\;\left(t=0\right)=0\;\text{and}\;\Theta_{th}^{i}\;\left(t=0\right)=0,$$

with zero mean

(39) $$\langle H_{th}^{i}\left(t\right)\rangle =0\;\text{and}\;\langle \Theta_{th}^{i}\left(t\right)\rangle =0.$$

The magnitude of the thermal fluctuations is encoded in the averaged variances reading:

(40) $$\langle H_{th}^{i}\left(t\right)H_{th}^{j}\left(t^{\prime }\right)\rangle =\frac{2\cdot k_{B}T}{\gamma_{0}\cdot \left|m\right|}\;\cdot \frac{1+\alpha^{2}}{\alpha }\cdot \delta_{ij}\delta \left(t-t^{\prime }\right),$$

(41) $$\langle \Theta_{th}^{i}\left(t\right)\Theta_{th}^{j}\left(t^{\prime }\right)\rangle =12\cdot k_{B}T\cdot \eta V_{H}\cdot \delta_{ij}\delta \left(t-t^{\prime }\right),$$

with the Cartesian spatial coordinates, *i*,  *j* ∈ *x*,  *y*,  *z*. The magnitude of fluctuations depends on MNP parameters, as well as the Boltzmann constant, *k*_B,_ and the temperature, *T*. Furthermore, the magnitude is unambiguously defined in 3D‐space (Kronecker–Delta function δ_*ij*_) and in time (Dirac–Delta function δ(*t* − *t*′)). The introduction of thermal fluctuations by inserting Equations (38) and (39) into Equations (36) and (37) yields a set of coupled stochastic differential equations that describes the nonlinear magnetization behavior over time in non‐equilibrium conditions for an arbitrary excitation field.^[^ ^12^, ^79^ ^]^ Solving these equations requires stochastic calculus schemes and numerical integration (whose details are out of the scope of this publication; please refer to references^[^ ^12^, ^59^, ^79^, ^86^ ^]^ for further details on how we implement the relaxation dynamics of MNP for simulation prediction). These dynamic relaxation simulations (DRS) mark the current state‐of‐the‐art for predicting the magnetic response of MNP to an arbitrary alternating magnetic field.

##### Verifying Applicability of Dynamic Relaxation Simulations

The DRS method described above has been extensively developed and applied in the field of biomedical applications: Among others, it has been used to estimate MNP‐tracer performance for MPI application,^[^ ^12^, ^86^ ^]^ to predict particle heating for hyperthermia therapy^[^ ^53^, ^79^ ^]^ and also extensively study the effect of MNP shape^[^ ^87^, ^88^ ^]^ and interaction on MNP relaxation.^[^ ^89^ ^]^ In recent years these initially solely in‐silico applications of DRS have been successfully used to match and interpret experimental results:, E.g., to correctly model the dynamic magnetization of MNP inside cells via simulating dynamic hysteresis loops,^[^ ^90^ ^]^ as well as MPI harmonics^[^ ^91^ ^]^ or to match the particle heating behavior in‐vitro.^[^ ^92^ ^]^ Most recently, also the generation of FMMD signals was successfully matched and analyzed using DRS^[^ ^74^, ^93^ ^]^ (further details are discussed in chapter 4 below). The DRS method is therefore considered sufficiently verified against experimental results to generate predictions for the nonlinear MNP behavior under non‐equilibrium conditions as presented recently.^[^ ^94^ ^]^

### 3.2 Adapting Approaches for Polydisperse MNP

**Figure 6:**
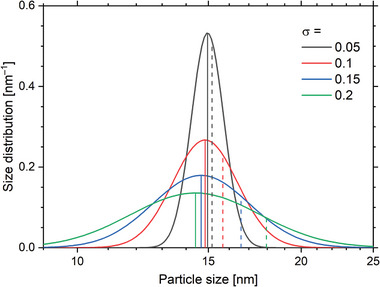
Lognormal size distributions with median *d*_0_ = 15 nm, for different width parameters σ on a logarithmic scale. The solid vertical lines mark the most probable values (denoted as mode), the dashes indicate the volumetric mean, i.e., the third moment of the distribution.

In reality, magnetic particles are not monodisperse but they exhibit a size distribution with significant width.^[^ ^95^ ^]^ Typically, the particle sizes obey a log‐normal distribution,^[^ ^96^ ^]^ as sketched in **Figure** **6**, whose probability density function (PDF) is mathematically expressed as:^[^ ^97^ ^][^ ^98^ ^]^

(42) $$f\;\left(d\right)=\frac{1}{\sqrt{2\pi }\sigma \;d}\;\exp\left[-\frac{\ln^{2}\left(d/d_{0}\right)}{2\sigma^{2}}\right]$$

With the median size *d*_0_ and the distribution width σ. By taking the derivative of Equation (42), it is found that the position of the maximum of the probability density is at *d*_0_ · exp (− σ^2^)_*max*_, i.e., shifted toward smaller sizes *d*, as marked by the solid vertical lines in Figure 6. The maximum value of the distribution is $\left(\;d_{max}=\frac{1}{\sqrt{2\pi }\;\sigma \;d_{0}}\;\exp\left(\frac{\sigma^{2}}{2}\right)\right)$. The arithmetic mean of the distribution is $\bar{d\cdot f(d)}=\int_{0}^{\infty }dd\cdot d\cdot f\left(d\right)=d_{0}\;\exp\left(\frac{1^{2}}{2}\sigma^{2}\right)$, the volumetric mean represents the third distribution moment and is peaking at $\bar{d^{3}\cdot f\left(d\right)}=d_{0}^{3}\;\exp\left(\frac{9}{2}\sigma^{2}\right)$. The short dashes mark the distributions’ volume mean. It should be noted that for wide distributions, the volumetric mean is strongly shifted to the distribution's large size tail because the large particles contribute most of the total volume.

Note that, whenever the lognormal distribution is used to describe or model an ensemble of MNP in the following, the mean core size of the particles is used as the median size of the distribution, i.e., *d*_0_ = *d_c_*. Instead of characterizing the width of the lognormal distribution by the dimensionless parameter *σ*, the quantity “polydispersity index” (PDI) is often used. In the Supporting Information, section A, the equations are given for calculating back and forth between the quantities.

#### 3.2.1 Implementation in Calculation under Equilibrium Conditions

The total iron oxide mass of the particle sample containing *n_p_* particles is calculated by the integral

(43) $$m_{FeO}=\rho_{FeO}\;\cdot n_{p}\cdot \frac{\pi }{6}\cdot \int_{0}^{\infty }dd_{c}\cdot f\left(d_{c}\right)\cdot d_{c}^{3}$$

with *ρ_FeO_* denoting the density of the core material. In case of a lognormal distribution according to equation (42), the integral can be evaluated, yielding an iron oxide mass of

(44) $$m_{FeO}=\rho_{FeO}\;\cdot n_{p}\cdot \exp\left(\frac{9}{2}\sigma^{2}\right)\cdot \frac{\pi }{6}\cdot d_{c}^{3}$$

The total magnetic moment *m_z_* is the sum of the moments of the individual particles, each of which is given by a Langevin equation, with individual particle's saturation moments *m_s_*_,_ *_i,_*

(45) $$m_{z}=\sum_{i}m_{s,i}\cdot L\left(\frac{m_{s,i}\cdot B}{k_{B}T}\right)\;$$

In a continuum formulation of Equation (45), the total magnetic moment is expressed by the integral over the particle size distribution times the number *n_p_* of particles,

(46) $$\begin{array}{cc} m_{z} & =\int_{0}^{\infty }dd_{c}\;\cdot f\left(d_{c}\right)\cdot n_{p}m_{s}\left(d_{c}\right)\cdot L\;\left(\frac{m_{s}\left(d_{c}\right)\cdot B}{k_{B}T}\right)\\ & =\frac{M_{s}\pi \;n_{p}}{6}\int_{0}^{\infty }dd_{c}\cdot f\left(d_{c}\right)\cdot d_{c}^{3}\cdot L\left(\frac{M_{s}\pi \;d_{c}^{3}B}{6k_{B}T}\right) \end{array}$$

For small excitation amplitudes, the total magnetic moment of such a lognormally distributed particle ensemble can be expanded to yield

(47) $$\begin{array}{cc} m_{z} & =\frac{M_{s}\pi \;n_{p}}{6}\int_{0}^{\infty }dd_{c}\cdot f\left(d_{c}\right)\cdot d_{c}^{3}\cdot [L\underset{\xi_{0}}{\underset{-}{\left(\frac{M_{s}\pi \;d_{c}^{3}B_{0}}{6k_{B}T}\right)}}\\ & \;+L^{\prime }\left(\xi_{0}\right)\frac{M_{s}\pi \;d_{c}^{3}}{6k_{B}T}\left(B-B_{0}\right)+\cdot \cdot \cdot ]\\ & =\frac{M_{s}\pi \;n_{p}}{6}\int_{0}^{\infty }dd_{c}\cdot f\left(d_{c}\right)\cdot d_{c}^{3}\\ & \;\cdot \left[\sum_{n=0}^{\infty }\frac{1}{n!}L^{\left(n\right)}\left(\xi_{0}\right){\left(\frac{M_{s}\pi \;d_{c}^{3}}{6k_{B}T}\right)}^{n}{\left(B-B_{0}\right)}^{n}\right] \end{array}$$

#### 3.2.2 Implementation in Simulations under Non‐Equilibrium Conditions

For particle relxation dynamics describing the nonlinear particle response under non‐equilibrium conditions (see section 3.1.3), the particle size distribution, Equation (42) is indirectly implemented as a pre‐condition for the core size and hydrodynamic size of the polydisperse MNP ensemble simulated: Typically, a minimum of 1000 particles is simulated,^[^ ^75^, ^86^ ^]^ in which each individual MNP follows the log‐normal distribution given above. This is also applied in this work. The effect on magnetization is then directly given by the size‐dependency of core and hydrodynamic volumes, *V_C_* and *V_H_*, in the coupled Equations (12) and (13), based on Equation (5). Every result of DRS discussed in the following thus naturally includes a core size‐distribution.

### 3.3 Comparison and Capabilities of the Modeling Methods

The modeling methods given above have individual regimes of application depending on their limitations weighted against their computational expenses. A brief overview of their methodological basis and limitations as well as application examples is summarized in **Table** **2** for convenient comparison and quick orientation. It aims to assist interested readers with a basis for deciding on what method to choose if own investigations are planned. All models consider polydisperse MNP distributions (see Section 3.2).

**Table 2:** Comparison of methods for modeling the FMMD signal as presented in this work.

	Linear and equilibrium;	Nonlinear and equilibrium;		Nonlinear and non‐equilibrium
Method:	Taylor expansion of Langevin function; equations	Langevin function derivatives; equations	Demodulated Langevin function	Dynamic Relaxation Simulations (DRS): Coupled relaxation equations;
Set of equations	(17) to (21)	(28) to (31)	(26) or (46)	(12) to (15) and (34) to (39)
Limited to:	Small amplitudes, *B_i_* ≪ 1 mT;	Small amplitudes, *B_i_* < 0.2 mT;	High(er) ampmlitudes, *B_i_* > 0.2 mT;	Superparamagentic regime, e.g. *d_C_* ≤ 30 nm for magnetite. Technically none, computational resources needed (and therefore potentially limiting)
Computational expenses	Low	Low	Low	High
Best applied for / examples	Estimate FMMD signal behavior in first approximation (s. Table 1); approximation of initial particle response to a dynamic excitation field with *B* < 0.1 mT;, e.g., for temperature^[^ ^99^ ^]^ and MNP concentration probing^[^ ^100^ ^]^	Reproduction of experimental results with B∼(0.1−10) mT with single input parameter dependency;, e.g., for size‐reconstruction of MNP from experimental FMMD results^[^ ^101^ ^]^ or probing MNP relaxation times using FMMD.^[^ ^102^, ^103^ ^]^		Predicting magnetic relaxation behavior of an ensemble of MNP in real time upon several input parameters;, e.g., field parameters and/or magneto‐physical properties of MNP for signal reconstruction of experimental data, probing size‐distributions of MNP from experimental data and predicting optimal MNP for signal generation in FMMD.^[^ ^74^, ^93^, ^94^ ^]^

#### 3.3.1 Exemplary Modeling of Field‐ and Size‐Dependency of FMMD Signals

In the following, we present exemplary results of FMMD signals obtained using the models above, focusing on the dependency of the applied AMF as well as the particle core size. This choice is based on the following assumptions: As seen from Equation (1), the magnetic response of MNP (magnetization) is directly dependent on the input field (Equation 16). Furthermore, the magnetic response of MNP to an AMF is directly dependent on the MNP core size, reaching higher overall saturation magnetization at the same absolute fields for larger sized MNP as demonstrated before in section 2.2 (Figures 5 and 4). It is therefore reasonable to understand that from all the intrinsic particle properties the MNP core size, i.e., the magnetic volume per particle, is the most dominant for biomedical applications as demonstrated in literature for MPI,^[^ ^31^, ^104^ ^]^ MFH^[^ ^92^, ^105^ ^]^ and biosensing applying FMMD.^[^ ^74^, ^94^ ^]^ By describing the combined effect of these two parameters on FMMD signal generation, the capability of FMMD in general as well as the model's usefulness for predicting the FMMD signal under realistic conditions shall be demonstrated. Finally, it will foster understanding of signal generation from the underlying fundamental principles already established above in chapter 2.

**Figure 7:**
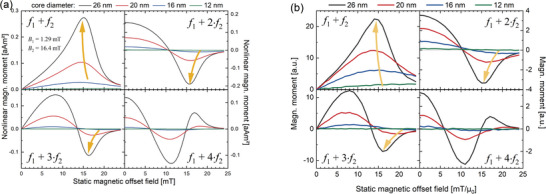
Nonlinear magnetic moment of MNP at mixing frequencies *f*_1_ + n · *f*_2_, with *n*  =  (1, 2, 3, 4) as a function of the static magnetic offset field *B*_0_ for quasi‐monodisperse (σ  =  0.05) magnetite particles of different mean core diameters *d*_C_ (color‐coded), calculated for *B*_1_ = 1.29 mT, *f*_1_ = 30.5 kHz, *B*_2_ = 16.4 mT and *f*_2_ = 63 Hz. (a)) is calculated from the demodulated Langevin function (Equation 46), while (b)) is derived from DRS. Violet semi‐transperent arrows are a guide to the eye, demonstrating peak‐shifts with changing particle sizes. Note that values in (b) are arbitrary and scaled to facilitate comparison of trends with (a)).

For exemplary modeling the FMMD signal in the following, the maximally achievable excitation field parameters of a typical magnetic reader were chosen with *B*_1_ = 1.29 mT at high frequency *f*_1_ = 30.5 kHz and *B*_2_ = 16.4 mT at low frequency *f*_2_ = 63 Hz.^[^ ^42^, ^106^ ^]^ In this way, we aim to describe the maximum signal possible under realistic conditions. The static magnetic offset field *B*_0_ varied from 0 to 25 mT, thereby probing different (non‐)linear responses while “scanning” the MNP magnetic response (as shown in Figure 4). Results derived from the demodulated Langevin model, and the DRS are plotted in **Figure** **7**a,b), respectively, to allow comparative evaluation of both models. The particles were modeled almost monodispersely with σ  =  0.05 to isolate the size effect on signal generation (as seen from Figure 6, the various sizes in a polydisperse sample would result in an overlapping size effect, diminishing the conclusiveness of our analysis).

From Figure 7a,b several implications on the signal generation in dual‐frequency excitation can be drawn: First, the measured nonlinear magnetic moment increases generally with increasing particle sizes for all four mixing frequencies analyzed. This is in accordance with the size‐dependent dynamic magnetization response of MNP, as there is generally more magnetic material to generate a signal, as discussed in section 2.2. Second, there is an ideal offset field for each core size that is maximizing the nonlinear magnetic moment in each of the four mixing frequencies. Interestingly, the position of this maximizing static field decreases for increasing core sizes, except for *f*_1_ + *f*_2_ derived from the demodulated Langevin function (discussed separately below). This general shift is explained by the increased slopes and combined faster arrival in saturation in the dynamic magnetization‐curves of MNP with increasing core sizes, which therefore generates the stronger signal at smaller offset fields as discssed above and summarized in Figure 4. Third, a similar finding of FMMD signal depending on the core size is made for the zero‐crossings of the magnetic moment in dependence of *B*_0_, which can be used for highly accurate sample detection (further discussed in applications, section 4.2.1). These findigs demonstrate that the FMMD signal can be fine tuned to detect particles of a specific size, which is the basis for size‐differntiation analysis (section 4.1 offers a detailed overview on this technique).

Last, comparing methods used in Figure 7, i.e., Langevin demodulation (a) versus DRS (b), a remarkable resemblance of the shapes of FMMD signals from both methods is apparent. The only qualitative divergence is the afore‐mentioned exception of the peak‐position in dependence of the offset field for *f*_1_ + *f*_2_ from demodulated Langevin functions, which shows a slightly increased offset field generating maximum signal with increasing core size.

## 4 Applications

### 4.1 Biomedical Applications

Established in 2007^[^ ^41^ ^]^ and thus being a relatively young technique among magnetic sensors, FMMD already finds numerous applications in biosensing, imaging and materials characterization. In the following, we generally summarize the latest findings and research approaches toward these applications. In specific, we first elaborate on the direct application of FMMD in biosensing (section 4.1.1) and second explain the fundamental similarity to the prominent biomedical applications of magnetic particle imaging (MPI) in section 4.1.2. Finally, we present an overview of (future) theranostic applications combining FMMD with other promising techniques and how hybrid platforms of these technologies could be realized (section 4.1.3). Finally, the fundamental understanding of the relationship between the relaxation process and the underlying physical and materials’ parameters of MNP (whose foundation is laid in chapter 2 above) allows to apply FMMD also for fast and precise materials characterization techniques (see section 4.2).

#### 4.1.1 Biosensing

MNP have a broad and diverse record for application as tracers and markers in biosensing,^[^ ^107^, ^108^, ^109^ ^]^ specifically ranging from intracellular magnetic tracers for detection and quantification by static‐field SQUID magnetometry,^[^ ^110^, ^111^ ^]^ markers in static/dynamic‐field magnetic resonance‐based detection of nucleic acids^[^ ^112^ ^]^ and proteins/pathogens,^[^ ^113^ ^]^ to dynamic field‐driven detection of specific affinity‐binding, e.g. for biotin‐streptavidin.^[^ ^114^, ^115^ ^]^ While the former techniques go beyond the scope of this review, the latter dynamic field‐driven technique is directly based on the alternation of Brownian relaxation behavior due to changes in the direct MNP vicinity^[^ ^27^, ^116^ ^]^ (and AC‐field parameters^[^ ^117^ ^]^). This change is either caused by specific affinity binding that increases the hydrodynamic radius (or volume) of MNP and thus changing Brownian relaxation behavior according to equation (10).^[^ ^57^, ^118^ ^]^ Or else, the relaxation behavior of MNP is changed due to clustering,^[^ ^119^ ^]^ as detectable by magnetic particles spectroscopy, MPS, (0D MPI, see section 4.1.2 below) down to detection limits of a few picograms of MNP as shown in matrix gels^[^ ^120^ ^]^ or inside cells.^[^ ^30^, ^121^ ^]^

As explained above in chapter 2, FMMD relies on this very processes of magnetic particle relaxation, independent of whether they are described as linear or nonlinear magnetic responses (s. section 3.1) and thus FMMD measurements qualify directly for detecting such change in MNP environment with increased flexibility from the frequency mixing terms: Usually, a sum‐frequency component *f*_1_  +  2*f*_2_ is analyzed for quantification of pathogens,^[^ ^122^ ^]^ as its zero‐crossing allows for a very sensitive and robustly detected characteristic feature. However, the component *f*_1_  +  *f*_2_ yields a stronger signal if a suitable static magnetic offset field is applied (as discussed in section 3.3.1, cf. Figure 7).

Consequently, FMMD‐based magnetic immunoassays (MIA) have been successfully applied to detect many small molecules like toxins to proteins, viruses, cells and bacteria:, e.g., the detection of C‐reactive protein (CRP),^[^ ^123^ ^]^ of *Francisella tularensis*,^[^ ^124^ ^]^ of *Yersinia pestis* ^[^ ^125^ ^]^ and of *Brucella spp*.^[^ ^122^ ^]^ was published recently. Furthermore the capability of FMMD to detect different influenza viruses was demonstrated,^[^ ^126^, ^127^ ^]^ with specific application to influenza A (H1N1).^[^ ^128^ ^]^ The detection of plant viruses,^[^ ^129^ ^]^ of Cholera toxin B^[^ ^130^ ^]^ of aflatoxin^[^ ^131^ ^]^ and of antibiotics in milk^[^ ^132^ ^]^ by FMMD has been reported. Also, recent developments demonstrated the feasibility of FMMD‐based detection of serological SARS‐CoV‐2 antibodies^[^ ^133^ ^]^ with direct application in wide‐ranged point‐of‐care diagnostics during future epidemics.^[^ ^134^ ^]^

With MNP of different properties, magnetic separation and labeling can be optimized.^[^ ^135^ ^]^ From this, Lab‐on‐Chip applications employing minimized reagent volumes and thereby minimized dosage are made possible.^[^ ^136^ ^]^ The method has been applied successful for in situ analysis of free radicals from the photodecomposition of hydrogen peroxide and is therefore presumably also applicable to other free radicals.^[^ ^137^, ^138^ ^]^ It is naturally highly specific to superparamagnetic particles’ key properties and allows the realization of multiparametric MIA in different matrices.^[^ ^123^, ^124^, ^125^, ^139^ ^]^ One such key property is MNP clustering, where the particles agglomerate due to broken or dissolved shells, increased (magnetic) particle‐particle interaction and/or other chemically enforced effects.^[^ ^37^, ^95^ ^]^ While discussion of whether clustering effects of MNP are generally amplifying^[^ ^120^ ^]^ or diminishing^[^ ^140^ ^]^ the magnetic relaxation signal is still ongoing in all field of medical applications of MNP,^[^ ^70^, ^141^, ^142^, ^143^ ^]^ single core, monodisperse MNP are of undisputed interest and potential for highly‐sensitive sensing applications.^[^ ^144^, ^145^, ^146^ ^]^ Recently, Rösch et al. proposed MPS‐based MIA that allow ultrasensitive quantitative detection by specific de‐clustering of MNP triggering a self‐enhancing signal‐amplification cascade, so called magnetic signal amplification circuit (MAC), allowing a fourfold increase in sensitivity.^[^ ^147^ ^]^ More complex applications even allow for MIA based on 3D printed column stacks,^[^ ^148^ ^]^ which rely on interpreting the FMMD phase signal during frequency scanning and allow multiplex detection of up to three different binding‐agents bound to suitable MNP systems of well‐differentiable core sizes.^[^ ^85^, ^149^ ^]^ Interestingly, the same principle is applied for multi‐channel MPI (whose details are explained in the next section 4.1.2), opening synergistic diagnostic methods combinations in the future.^[^ ^150^ ^]^

#### 4.1.2 Imaging and Spectroscopy

The magnetization relaxation mechanism of MNP in a sinusoidal magnetic field as explained in section 2.1.2 can be generally exploited for direct (positive contrast) imaging in the magnetic particle imaging (MPI) technique^[^ ^151^ ^]^ and has been intensively researched for diagnostic application in the past decade.^[^ ^152^, ^153^, ^154^ ^]^ The MPI principle is equivalent to that of FMMD limited to one excitation field with typical field amplitude *B*_0_ ≈ 20 mT and frequencies of f∼(10−25) kHz.^[^ ^155^ ^]^ MPI measures the derivative of the nonlinear magnetization, *M*′ (*t*) =  *dM*/*dH* (equation (13)), which will be zero, when a sufficiently high offset field, *B*_0_, saturates the MNP magnetization, and non‐zero, where *B*_0_ =  0. In MPI, the sample is therefore exposed to a saturating offset field ($B_{0}∼1$ T), while simultaneously a field‐free point with *B*_0_ =  0 is scanned across the sample volume, allowing to localize MNP by their nonlinear relaxation response to the single‐frequency excitation field. This process allows 2D and – in principle also – 3D mapping of the direct MNP distribution across the sample.^[^ ^156^, ^157^ ^]^ Translating the spatial image to frequency space, MPI can equally be expressed as a recording of the higher harmonics of the nonlinear magnetization response of MNP. These harmonics are also commonly used for application in MIA via so‐called magnetic particle spectroscopy (MPS),^[^ ^33^, ^158^ ^]^ which is basically a 0D MPI scan. Please note that these harmonics are directly dependent on the particles nonlinear relaxation behavior (see. section 2.1.2), MPI/MPS has been successfully applied to detect a variety of materials parameters, as will be detailed in section 4.2 below.

Advantageously compared to MPI, FMMD does not require such strong offset field for saturating the MNP (see section 3.3.1), making FMMD potentially much more cost‐efficient and portable, while not being limited in the lateral (*x* − /*y* −) dimensions.^[^ ^159^ ^]^ Indeed, the FMMD method has been successfully applied by Hong et al. to generate MPI‐like images by using a planar (p‐)FMMD setup consisting of twined measurement heads setup with identical but inversely wound coils.^[^ ^160^ ^]^ Halbach magnet arrangements can be employed to further improve imaging quality.^[^ ^161^ ^]^ The p‐FMMD setup has been furthermore reported to successfully serve well for imaging the distribution of MNP from 3D‐rendering of 2D tissue slices of a rat brain^[^ ^162^ ^]^ as well as an MIA platform for combined application.^[^ ^163^, ^164^ ^]^ Recently, the aforementioned successes amounted into the breakthrough demonstration of a p‐FMMD‐based MPI device for the point‐of‐care diagnostics, which is portable, energy‐efficient and able to detect MNP amounts down to a limit of detection of 1.3 µg(Fe).^[^ ^165^ ^]^ The device has furthermore been successfully tested for imaging the biodistribution during preclinical small animal trials in mice, employing CT26 and MC38 tumor models.^[^ ^166^ ^]^

**Figure 8:**
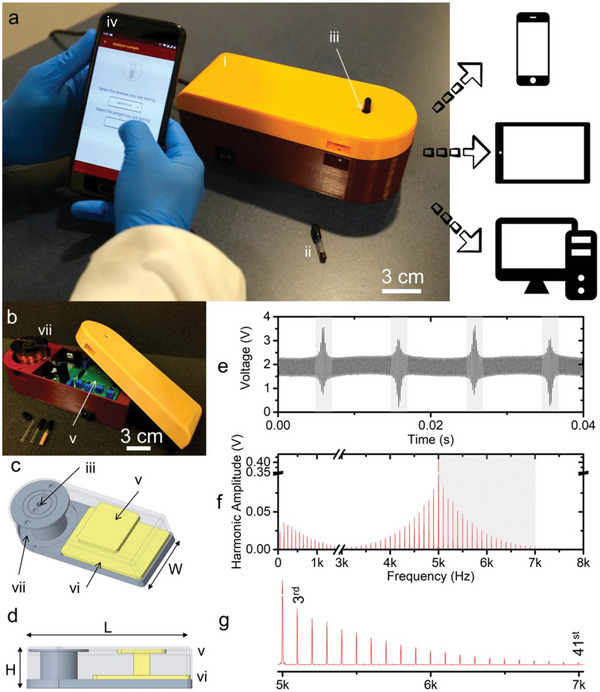
(a) Photograph of the MagiCoil portable device with a smartphone application. The overall dimensions of the device are 212 mm (L) × 84 mm (W) × 72 mm (H). (i) Device shell is 3D printed using the material. (ii) Disposable, USP type I glass vial containing the MNP sample. (iii) Sample loading port. (iv) Smartphone application. (b) Photograph of the internal structures of the MagiCoil device. (c) 3D model of the MagiCoil device with (v) top and (vi) bottom circuit boards, and (vii) three sets of copper coils for generating magnetic driving fields and collecting dynamic magnetic responses of MNPs. (d) Side view of the 3D model with length (L) and height (H) labeled. (e) Discrete time voltage signal collected from pick‐up coils during two periods of low‐frequency field. The dynamic magnetic responses of MNPs cause visible spikes as highlighted in gray regions. (f) Frequency domain MPS spectra from (e). Higher harmonics are observed. (g) Enlarged view of higher harmonics (the 3rd to the 41st harmonic) between 5 and 7 kHz. Reproduced under terms of the CC‐BY license with permission from K. Wu et al., ACS Applied Materials & Interfaces 13, 7966.^[^ ^102^ ^]^ Copyright 2021 American Chemical Society.

For future applications, the sensitivity of FMMD (and MPS alike) could be further enhanced by hardware optimization, e.g. by the very recently proposed frequency‐selective signal amplification using a passive receive coil setup.^[^ ^167^ ^]^ On the other hand, the MNP tracers can be tuned, with frequency optimized core sizes^[^ ^168^, ^169^ ^]^ or effective magnetic anisotropies,^[^ ^170^, ^171^ ^]^ which match the applied FMMD field parameters ideally (see also section 4.1.3; second to last paragraph). As of late, MPS also experiences a rapidly growing interest as a low‐cost‐easy‐to‐apply technique for point‐of‐care diagnostics.^[^ ^172^, ^173^ ^]^ In fact, Wu et al. have already proposed in 2021 that low‐cost, portable, easy‐to‐handle (dual‐frequency) MPS devices are feasible when introducing the MagiCoil.^[^ ^102^ ^]^ As demonstrated in **Figure** **8**, this handheld device is capable of generating FMMD signaling (cf. Figure 3) and differentiates up to 20 harmonics in MPS (Figure 8 (e) through (g)). It operates flexibly via suitable app‐interfaces (e.g., via smartphone or tablet), thus enabling first‐approach MNP characterization and ‐even more promising – preliminary point‐of‐care immunoassay diagnostics in remote situations or less developed infrastructures. Transferring FMMD from (specialized) lab scale to (generalized) easy‐to‐handle applicability in the field is expected by Yari et al. to inspire further optimization potential in the future.^[^ ^164^ ^]^

#### 4.1.3 Toward Theranostics

The term *theranostics* combines elements of therapy and diagnostics in interventional medical treatment:^[^ ^174^ ^]^ A combination of (imaging) diagnostics (e.g., magnetic resonance imaging, PET/SPECT, near‐infrared spectroscopy and biosensing) and therapy options (e.g., radio‐and chemotherapy, gene therapy/ delivery and hyperthermia) is applied simultaneously to allow advanced personalized medical treatment.^[^ ^175^, ^176^ ^]^ A prominent example is the field of nuclear medicine, more specifically the application of radioactive substances for cancer localization and therapy, e.g., for thyroid cancer with radioactive iodine.^[^ ^177^, ^178^ ^]^ The small size and biocompatibility of iron‐oxide MNP as well as their broad and versatile biomedical application naturally qualifies them as suitable candidates for the use as theranostic agents.^[^ ^179^, ^180^, ^181^ ^]^

The biosensing and imaging applications cover the diagnostic elements and the combination of FMMD and MPI was already discussed (section 4.1.2). Therapeutic effects are readily available from MNP as heating agents for hyperthermic treatment in MFH.^[^ ^59^, ^182^, ^183^ ^]^ By applying excitation frequencies of (50 − 1000) kHz at field amplitudes of (10 − 50) mT, MNP and their direct vicinity can experience a local temperature increase by up to ten degrees Celsius and above,^[^ ^184^, ^185^, ^186^ ^]^ which is used either for direct cell damage^[^ ^187^, ^188^, ^189^ ^]^ or localized drug release from thermos‐sensitively functionalized particle shells.^[^ ^181^, ^190^ ^]^ Indeed, (MPI‐)image‐guided accumulation and subsequent therapeutic heating of MNP has been intensively researched over the past years.^[^ ^191^, ^192^, ^193^ ^]^ In detail, the following remarkable successes are noteworthy:

- In 2017, Hensley et al. reported on the first simultaneous application of MPI and MFH in *vitro* with one single device; demonstrating feasible MPI (at *f*  =  20 kHz and *B*  =  20 mT) in combination with heating rates of up to 0.4 °C/s (i.e., 150 W/g(Fe) at *f*  =  354 kHz and *B*  =  20 mT) using nanomag‐MIP SPIO (from micromod Partikeltechnologie GmbH; varying core sizes from 20 to 100 nm).^[^ ^194^ ^]^
- In 2018, Tay et al. reported on first‐time in‐vivo MPI‐guided heating using the same device as above, demonstrating localized heating of 120 W/g(Fe) for MFH conditions (s. above) within an area of the “size of a grain of rice” (1–2 cm) using 13 nm core‐sized MNP but no temperature rise elsewhere and otherwise for MPI conditions (s. above).^[^ ^195^ ^]^
- In 2020, Wells et al. demonstrated first‐time in‐vitro MPI‐MFH feasibility using Lissajous trajectories^[^ ^196^ ^]^ (commonly used trajectory that strongly improve the prediction rates for MPI image reconstruction).
- In the same year, Lu et al. finally reported on a first commercially available MPI‐MFH scanner (from Magnetic Inside Inc., USA).^[^ ^197^ ^]^
- In 2022, Healy et al. identified that successful clinical translation of MFH inevitably requires simultaneous MPI for accurate localization and thermometry and listed three main challenges: a) tolerability of the human body to the applied EM‐fields, b) large enough EM‐fields to engulf body volume (e.g., entire torso), i.e., requiring large coils of low power consumption and c) suitable MNP systems ideally optimized for MPI, MFH and thermometry performance at the same time.^[^ ^192^ ^]^
- In 2023, Behrends et al. put forward the idea to use (stereolithographic) additive manufacturing to accommodate auxiliary devices to MPI systems and demonstrated feasibility for a MPI‐MFH theranostic platform design.^[^ ^198^ ^]^
- Just recently, in 2024, Buchholz et al. introduced a MFH‐insert to transform a commercial MPI to a hybrid theranostic platform and demonstrated general tomographic MPI capability combined with local MFH (heating selectively limited to 8 mm cubical) and multicolor thermometry with an in‐vitro phantom.^[^ ^199^ ^]^

Interestingly, applying FMMD instead of MPI for a theranostic platform addresses all of the three challenges listed by Healy et al. (2022) above:^[^ ^192^ ^]^ p‐FMMD removes challenge b) directly by being spatially unlimited, as detailed out in section 4.1.2 above. Furthermore, Liu et al. demonstrated nano‐thermometry on the basis of nonlinear magnetization of MNP with up to 0.1 K accuracy,^[^ ^200^ ^]^ which is technically also readily available using FMMD (s. section 2.2 above). Finally, a medically tolerable EM‐field exposure can be quantified by the product of frequency and field strength,^[^ ^201^ ^]^ $f\left[\frac{1}{s}\right]\cdot H\left[\frac{A}{m}\right]$, for which the International Commission on Non‐Ionizing Radiation Protection (ICNIRP) lists as a limit of exposure for humans of *f* · *H* ≤ 8 · 10^8^ A/(m · s).^[^ ^202^ ^]^ This limit can be used to appropriately address challenge a), allowing, e.g., a combination of *f* ≤ 80 kHz with *H* ≤ 10 kA/m (*B* ≈ 12.6 mT), well within FMMD and MPI as well as low‐field MFH requirements.

In the following, we discuss a potentially new theranostic platform combining FMMD with MFH and MPI based on the afore‐mentioned successful development of MPI‐MFH theranostics. An imaginable approach could be tracking the particle distribution in a subject with MPI, assessing the local particle concentration and binding state at point of interest with FMMD and finally generating heat with MFH to either release drug (scenario A) or apply therapeutic heating (scenario B). Specific binding of MNP to a target region, e.g. tumor cells or antibodies (for inflammatory regions) can be used to increase targeting efficacy and cellular update of MNP.^[^ ^203^, ^204^ ^]^ With FMMD quantitatively assessing the accumulated amount of MNP locally present and qualitatively identifying their binding state more sensitively than MPI from the analysis of intermodulation (see chapter 3), the two scenarios given above could concretely benefit as follows: In scenario A, more accurate quantification of MNP‐drug complexes delivered at (i.e., bound to) the micro‐structure of interest (tissue, cells, etc.) allows to lower the dosage of drug‐MNP complexes systematically administered in the first place^[^ ^205^, ^206^ ^]^ and to advance predictive models for drug delivery treatment planning.^[^ ^207^ ^]^ In scenario B, knowledge on the time‐resolved amount of MNP internalized inside tumor cells enables to enhance MFH efficacy by specifying the timing for applying the heating magnetic field (f ≈100 kHz), as heating on the nano‐scale inside cells dominates MFH effectiveness,^[^ ^187^, ^189^ ^]^ while cellular uptake of MNP itself is time‐ and cell type‐dependent.^[^ ^121^, ^208^ ^]^ To the best of the authors’ knowledge, no such attempts have hitherto been reported, most probably due to the relative juvenility of both FMMD and MNP‐based theranostics. However, as outlined before, the applications of FMMD,^[^ ^41^ ^]^ MPI^[^ ^12^ ^]^ and MFH^[^ ^59^ ^]^ all rely intrinsically on the same underlying magnetic relaxation physics. Consequently, also their combined theranostic application may build on the same physical foundations and its advancement may focus on the same dependencies and can be investigated on a simulative predictive basis with the framework described in this review.^[^ ^209^ ^]^ These theoretical predictions allow to address two dominant challenges for theranostic application of FMMD‐MPI‐MFH which are

- tuning the experimental (field) parameters to the most suitable combination available and
- identifying the ideal MNP (tracer) characteristics, indirectly requiring the MNP properties to remain reasonably producible at least at lab‐scale.

Considering challenge a) requiring suitable field parameters, FMMD and MPI agree with each other, typically ranging from $f_{2}∼\left(0.5-4\right)$ kHz and $B_{2}\;∼\left(5-20\right)$ mT for the drive and $f_{1}∼\left(20-50\right)$ kHz and $B_{1}\;∼\left(1-3\right)$ mT for the excitation field of FMMD and $f_{MPI}∼\left(10-25\right)$ kHz at field amplitudes of $B_{MPI}∼\left(10-20\right)$ mT for MPI. Tay et al. reported on optimal MPI‐imaging conditions for 27 nm MNP at *f*  =  1 kHz and *B*  =  14 mT,^[^ ^210^ ^]^ which suggest directly matching field parameters for the low‐frequency FMMD field and typical MPI setups. For MFH, however, strong fields (B∼(15−50) mT and f∼(100−1000) kHz) are required to generate reasonable heating rates, which are outside of the operational range of nowadays FMMD setups.^[^ ^211^ ^]^ However, simulative approaches predict optimized heating rates for large MNP (core size 25 nm and above) for *f* ≈ 50 kHz and *B* ≈ 15.8 mT,^[^ ^92^ ^]^ which are in agreement with low‐power photothermal heating of MNP^[^ ^212^, ^213^ ^]^ and thus within FMMD capabilities (under the precondition that FMMD and MFH could be applied consecutively in such a setup). Another very promising alternative chance for theranostic application of FMMD and MFH arises from low frequency (f∼10kHz and below) generated mechanical damage dealt to cells from physical rotation of membrane‐bound MNP:^[^ ^214^, ^215^ ^]^ this mechanism has been demonstrated by Zhang et al. to lead to apoptosis in INS‐1 cells applying frequencies of *f* ≈ (5 − 20) kHz to membrane‐bound (20‐100) nm core size BNF MNP.^[^ ^216^ ^]^ Furthermore, Mansell et al. showed that U87 brain tumor cells were reliably killed by membrane rupture due to slow rotation (*f* < 1 kHz) of membrane‐bound 2 µm CoFeB/Pt microparticles.^[^ ^217^ ^]^ Such a mechanical rupture also smartly avoids yet another challenge in in‐vivo application of MFH, as the heating capacity of MNP is known to decrease upon intracellular binding by approx. one third.^[^ ^218^ ^]^ These besides‐the‐common MFH applications clearly show that a combination of MFH and MPI/FMMD is feasible in the field parameter range of FMMD.

Considering the challenge b) of identifying most suitable MNP properties, the situation is more complex, as the of physical and magnetic properties of MNP are interdependent on each other and furthermore their interplay is not fully understood yet.^[^ ^219^ ^]^ Nevertheless, it is generally agreed that for Fe_3_O_4_‐MNP large core sizes between 20 and28 nm lead to improved signal intensities in FMMD,^[^ ^93^ ^]^ improved resolution and image quality in MPI^[^ ^25^, ^104^ ^]^ and increased heating in MFH.^[^ ^92^, ^220^ ^]^ The state of MNP, however, is yet under discussion: While, on the one hand, nearly monodisperse MNP formulations are available specifically for optimized MPI application^[^ ^221^ ^]^ and narrow‐size‐distributed, monodisperse MNP show improved signal quality in MRI, MPI and MFH.^[^ ^222^, ^223^, ^224^ ^]^ On the other hand, there is also evidence for improved performance of agglomerated MNP both for MPI and MFH^[^ ^120^, ^225^, ^226^ ^]^ applications. Also, multicore MNP are considered by some as superior for MPI and MFH applications,^[^ ^227^, ^228^ ^]^ while other strictly favor single‐core MNP.^[^ ^229^ ^]^ Besides core size, the relaxation behavior of MNP is also highly dependent on their effective magnetic anisotropy,^[^ ^230^, ^231^ ^]^ which in turn strongly depends on the interparticle magnetic dipole‐dipole interactions (ppIA).^[^ ^232^, ^233^ ^]^ The exact impact of interparticle interactions are a topic of ongoing complex discussion in the research community, e.g. considering MFH performance, Das et al. argue that stronger ppIA directly decrease the MFH performance,^[^ ^234^ ^]^ while Landi et al. argue both ways depending on the magnetic properties of MNP^[^ ^235^ ^]^ and Branquinho et al. assume that increased ppIA will automatically increase MNP effective anisotropy leading to chain formations,^[^ ^236^ ^]^ which again Saville et al. assume to increase the MFH performance.^[^ ^237^ ^]^ The latter example may serve to demonstrate the complexity of the ongoing discussion and explain why we will exclude further discussion on that topic from this review. Interested readers are referred to further general overview given in references^[^ ^232^, ^233^ ^]^ and theoretical descriptions in reference.^[^ ^238^ ^]^

Finally, we would like to comment on the commonly cited advantageous effects of magnetically guided targeting (MDT), which can– in theory –further enhance theranostic application.^[^ ^239^ ^]^ Its practical use must be viewed very critically, however, as an recent study by Wilhelm et al. analyzed over 100 independent experimental targeting studies and cast doubt on the practical relevance MDT by revealing a mean deposition of less than 1 % of the administered MNP at the desired site.^[^ ^240^ ^]^ As mentioned above, applying FMMD as a sensitive detector for MNP accumulation at a specific target area accompanied with knowledge on specific binding ratios of MNP from interpreting their individual intermodulation signal shows promise to improve this number. A detailed discussion goes beyond the scope of this review but can be continued generally with Venugopal et al.^[^ ^241^ ^]^ or specifically for dosing of MNP in MFH with Southern et al.^[^ ^242^ ^]^ or applicability of MFH in‐vivo with Rubia‐Rodríguez et al.^[^ ^243^ ^]^

### 4.2 Materials Characterizations

MNP characterization techniques are typically involving multi‐stage analysis with various methods such as diffraction and scattering techniques,^[^ ^244^, ^245^ ^]^ dynamic light scattering for hydrodynamic size,^[^ ^246^, ^247^ ^]^ electron microscopy for physical core size analysis^[^ ^248^ ^]^ and extraction of magnetic parameters from magnetization measurements (SQUID or VSM)^[^ ^249^ ^]^ and magnetic core size distribution using Chantrell‐fitting^[^ ^51^ ^]^ and ACMS.^[^ ^250^ ^]^ In comparison to these characterization methods, dynamic magnetic spectroscopy methods such as MPI/MPS and FMMD measure harmonics, which are directly dependent on the particles nonlinear relaxation behavior (see section 2.1.2) and thus provide a multitude of information on MNP with just a single measurement. Using these harmonics as a MNP systems characteristic “fingerprint”, MPI/MPS has been already successfully applied to detect a variety of parameters, including local temperature^[^ ^251^, ^252^ ^]^ and viscosity,^[^ ^253^, ^254^ ^]^ state of particle mobility/binding,^[^ ^251^, ^255^ ^]^ to distinguish materials and the degree of interparticle interactions,^[^ ^91^, ^256^, ^257^ ^]^ as well as core and hydrodynamic particle sizes^[^ ^104^, ^258^ ^]^ and (preliminary) cell viability assessment.^[^ ^259^ ^]^

The afore‐mentioned parameters can furthermore be measured quantitatively, e.g. as demonstrated for temperature^[^ ^260^ ^]^ and viscosity,^[^ ^261^ ^]^ or even simultaneously in a single MPI/MPS measurement^[^ ^262^, ^263^, ^264^ ^]^ using multi‐channel (also *multicolor* or *multiplex*) detection. As such, multi‐channel detection relies on the ability to differentiate the signals of the respective MNPs parameters and thereby track each parameter in an individual channel. An intuitive example demonstrating the potential of such multi‐channel detection was recently given by Shasha et al., who succeeded in the color‐coded differentiation and localization of three monodisperse MNP samples with different core sizes ranging between 23.8 nm, 25.2 nm and 27.7 nm with a resolution of 1 mm.^[^ ^258^ ^]^ Here, the mechanism enabling such precise differentiation via the harmonics was the highly specialized MNP tracer with a very narrow size‐distribution.^[^ ^104^ ^]^ Naturally, FMMD offers a direct advantage in multi‐channel detection by not only detecting the higher harmonics of the excitation frequency, $f_{1},\;f_{2},\text{…},f_{k}$, but also the sum‐frequency components, $f_{1}+f_{2},\;f_{1}+2f_{2},f_{1}+3f_{2},\text{…}$, as explained above (chapters 2 and 3). This allows defining several characteristic features unique to FMMD, as listed in Table 1 (section 3.1.1) above and will be exemplified further in section 4.2.1 below. Thus, dual‐frequency excitation provides additional information available for multi‐channel detection, whose feasibility has already been demonstrated for dual frequency MPS^[^ ^255^, ^265^ ^]^ and multiplex tracer detection with FMMD.^[^ ^85^, ^149^, ^266^ ^]^

In the following subsections, we will exemplify the principle of extracting additional information from FMMD signals using their characteristic magnetically detection features (cf. section 3.1.1) with focus on particle core size‐extraction in section 4.2.1 as well as touch base with two current examples of FMMD sample feature extraction techniques from FMMD measurements in section 2.4.2, which is a topic of ongoing research interest in the field as a cost‐and‐time‐efficient characterization technique for MNP.

#### 4.2.1 General Example: Characteristic Feature Extraction from FMMD Measurements

As discussed already, FMMD allows a more sensitive signal interpretation due to the additionally measured intermodulation terms, i.e., frequency mixing terms *f*_1_ + *nf*_2_. Concretely, there are several characteristic features to be accessible: Their maximum and minimum (peak) positions and their zero‐crossings (for higher terms, *n* ≥ 2). To demonstrate the capability of FMMD for materials analysis, we deliberately chose to demonstrate the characteristic feature extraction for a lognormally distributed ensemble of small particles with mean core size *d_C_* =  12 nm, which is generating an overall low intensity signal (as shown and discussed in section 3.3.1, Figure 7) – this however has no impact on FMMD signal interpretation, as demonstrated in this section.

**Figure 9:**
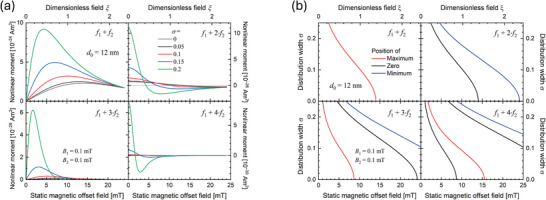
Exemplary characteristic feature extraction from FMMD signal of a lognormally distributed ensemble of magnetite MNP with mean core size *d_c_* =  12 nm in dual‐frequency excitation with *B*_1_ =  0.1 mT, *f*_1_ =  1260 Hz, *B*_2_ =  0.1 mT, *f*_2_ =  63 Hz, derived from Langevin function demodulation modeling (section 3.2.1, Equation 47). (a) Nonlinear magnetic moments per particle of the first four mixing frequencies for varied static magnetic offset fields *B*_0_ and different distribution widths σ. (b) Develelopment of the FMMD characteristic features for maximum (peak) position, zero‐crossing and minimum position as a function of the distribution width σ for the first four mixing frequencies (directly extracted from (a)). Note that the dimensionless field variable *ξ* (Equation 7) is shown as the top axis in both figures.

**Figure** **9** shows the average nonlinear magnetic moment per particle (mean core size 12 nm) for various distribution widths *σ *calculated from integrating Equation (47) for the first four mixing components as a function of the static magnetic offset field *B*_0_ up to 25 mT. The excitation and drive field amplitudes are chosen small *B*_1_ = 0.1 mT and *B*_2_ = 0.1 mT, so that the selection of the response of MNP to FMMD is dominantly governed by the offset field (cf. Figure 4a,b) and $\frac{f_{1}}{f_{2}}=20$ with *f*_2_ =  63 Hz.

The shape of the first three mixing terms in Figure 9a is comparable to that generated for larger field amplitudes, *B*_1_ and *B*_2_, in Figure 7, confirming the generality of the approach. For larger σ values, when the core size distribution becomes wider, the second maximum in the fourth mixing term is not distinguishable anymore because the negative responses from larger particles cancel the positive responses of smaller ones.

Note that for analysis of other core diameters, the dimensionless field variable *ξ* (Equation 8) is included in Figure 9. In fact, the dimensionless *ξ*‐scale remains unchanged, making Figure 9 a type of master‐curve for predicting an MNP particle ensemble's response to FMMD probing. This is because the size‐dependency cancels out of $\xi \propto d_{C}^{3}\cdot B\left(B_{0},B_{1},B_{2}\right)$, as the offset field scales with inverse particle core volume, $B_{0}\propto d_{C}^{-3}$. The curves for other diameters *d*_C_ therefore follow the same shape and are just scaled differently on both axes. For example, with larger particles, the features shift to lower magnetic offset field values but higher magnetic moment responses due to the afore‐mentioned size‐and‐field‐dependent signal generation of FMMD based on the nonlinear magnetization response as explained in section 2.2 (cf. Figure 4c) and as discussed in section 3.3.1.

The FMMD signal intensity, i.e., nonlinear magnetic moment of each particle increases strongly for wider distribution width, σ. This is caused by the existence of even larger particles in the ensemble (cf. size distribution, Figure 6), contributing much stronger to FMMD signal generation (cf. size dependency of FMMD, Figure 7).

The dominant signal contribution from the large size tail of the size distribution also leads to the observed shift of the position of characteristic features toward lower offset fields with increasing σ in Figure 9b. Among these, the zero‐crossings in the signals are of particular experimental relevance are, as they can be detected quickly and reliably: Since they are in fact 180°‐phase reversals of the signal, they are easily identified as a sign flip in a measurement for higher frequency mixing components *f*_1_ + *n*·*f*_2_, *n* = 2, 3, 4.

In terms of applicability, the detection of zero‐crossings in FMMD allows to determine at which offset field value these higher terms vanish for a certain mean core size *d*_C_ and size distribution width *σ*. Thereby, a higher detection precision than for the extreme values can be achieved in FMMD. This also allows for indirect extraction of the core size distribution of an ensemble of MNP from FMMD measurements, i.e., the parameters of mean core size and distribution width, under the condition of complementary knowledge about the sample. This is further elaborated and applied in the next section 4.2.2.

Additionally, Figure 9b reveals another characteristic feature of the fourth mixing component, *f*_1_ + 4·*f*_2_, where two zero‐crossings are detectable but with a distinctively different dependence on particle distribution width and applied magnetic offset field. Thus, a distinction of the two size parameters *d*_0_ and *σ* from the measured field dependence of, e.g., the mixing signal becomes feasible directly from the FMMD measurement. However, one must keep in mind that the fourth mixing term has low signal intensity and thereby needs a very sensitive recoding system or large enough quantity of MNP for reaching the detection limit (cf. Figure 5). The technique for analyzing higher frequency mixing components will be also applied in a different context in section 4.2.2.

#### 4.2.2 Specific Example: Extracting Particle Core Sizes from FMMD Measurements

The great sensitivity for magnetic particle spectroscopy for different particle properties in interplay with the chosen field amplitudes, as discussed in the foregoing sections of this chapter, is also it “Achilles heel” at the same time: the individual particle properties’ influences on the FMMD signal are superimposed in the measurement and individual extraction is challenging due to the complex interplay of dependencies. To handle this challenge, one needs to focus on a specific property to extract and use resulting complementary assumptions to narrow down the analysis of the FMMD signal. Since it is generally agreed that core size of MNP is their most influential intrinsic parameter and well understood (as discussed in sections 2.2 and 3.3.1), recent advances in MNP materials characterization using FMMD focus on extracting the particle core size distribution data. The principle is based on the understanding that the chosen field amplitudes open a “corridor of influence” from which to detect the MNP ensemble's dynamic magnetic response, which is size‐dependent (cf. Figure 4). Here, Pourshahidi et al. were able to demonstrate that by using a complementary iron‐content measurement, the particle core size distribution of MNP systems can be calculated unambiguously by demodulating the Langevin function applied to a single FMMD measurement^[^ ^101^ ^]^ and thereby exploiting the core size‐dependent, nonlinear magnetization response of MNP as principally discussed above (cf. Figure 4 (principle) and Figure 9b (direct application)). The method has been verified with several commercially available and well‐characterized MNP from Micromod Partikeltechnologie GmbH.^[^ ^267^ ^]^ However, this approach remains restricted, as the core size distribution cannot be extracted directly from fitting but must be approximated by fitting the experimental FMMD signal: Only by backfitting modeled results to the experimental data in recursion to refine best‐fitting size‐distribution parameters, also the core size distribution width can be extracted, as shown recently.^[^ ^93^ ^]^ Thereby, the method is not stand alone and retains a certain intrinsic uncertainty by relying on complementary measurements and subsequent fitting routines in the post‐processing of results.

**Figure 10:**
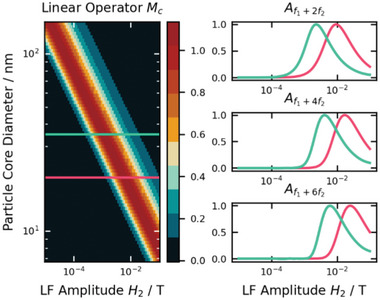
Modeled particle response to drive field amplitude scans. (a) Numerical mapping of the normalized *f*_1_ + 2*f*_2_ intermodulation component when scanning the drive field amplitude, *B*_0_, for different of the core size. (b) Exemplary intermodulation components for *f*_1_ + *nf*_2_, *n*  =  2,  4,  6 for mean core sizes *d_c_* =  20 nm (red) and *d_c_* =  35 nm (green). All intermodulation components are normalized to each individually scanned *B*_2_‐scan. Reproduced under terms of the CC‐BY license with permission from T. Bikulov et al., Int. J. Magn. Part. Imaging 10, 2 403 014.^[^ ^269^ ^]^ Copyright 2024 Infinite Science Publishing GmbH.

Very recent a different approach was proposed by Bikulov et al. demonstrating the feasibility to reconstruct the MNP core size distribution directly from FMMD measurements by probing the size‐and‐field‐dependency of FMMD (cf. Figure 4) by varying the drive field amplitude *B*_2_ at very low drive field amplitudes, $f_{1}∼10\;\mathrm{Hz}$ ^[^ ^268^ ^]^ at no offset‐field, *B*_0_ =  0: Under the condition that the energy in the system is dominantly sourced by the drive field, i.e., *B*_1_ · *f*_1_ ≫ *B*_2_ · *f*_2_, the intermodulation magnetic particle moment can then be described in terms of a passive mixing model superpositioning Bessel functions in a Hilbert transform‐based model of the Furier‐transformed Langevin function (allowing to arbitrarily model the input FMMD signal and modulated output signal analysis, and vice‐versa). In this way, the nonlinear magnetic response of MNP is probed in dependence of the drive field amplitude, *B*_2_, revealing indirect dependence of the even frequency mixing components on the mean core size *d_C_* and distribution width σ. These particle size‐distribution parameters are accessible by reconstruction that solves the linear inverse problem of mapping the mixing components signal to the respective core sizes’ contribution (further details are found here^[^ ^269^ ^]^). As depicted in **Figure** **10**, this mapping with a linear operator reveals a clear dependence of the particle core size to the applied drive field amplitude (Figure 10a).

In fact, according to this approach the sizes of interest for superparamagnetic particles, $d_{c}∼\left(10-30\right)$ nm are peaking with their signals for drive fields of $B_{2}∼\left(1-10\right)$ mT. Also, the FMMD signal peaks in the even intermodulation terms are clearly size‐dependent and peak intensity shifts to larger field amplitudes for higher terms, as seen from Figure 10b. By analyzing different intermodulation terms independently, e.g. *n*  =  2,  4,  6 as given here, multi‐channel (multi‐core) analysis is possible with the FMMD technique simultaneously. This is advantageous, since the differentiation of peaking intermodulation terms can be traced as sensitively as the drive amplitude can be selected. First validation to experimental data has been presented and is currently being expanded to evaluate hydrodynamic particle diameters.^[^ ^269^, ^270^ ^]^

Note that this approach of drive field amplitude scans, *B*_2_, is intrinsically limited to dual‐frequency excitation, as the core size‐dependence on *B*_2_ is present only in the even intermodulation terms, starting with *f*_1_ + 2*f*_2_.

## 5 Conclusion

In this work, we have presented the fundamental descriptions of the dynamic behavior of magnetic nanoparticles subjected to a dual frequency alternating magnetic field and provided an overview of its applications in diagnostic and therapeutic biomedicine as well as nanomaterials characterization. Several state‐of‐the‐art frameworks based on Langevin modeling calculations and stochastic Néel‐Brownian relaxation model simulations for describing the FMMD signal generated by mono‐ and polydisperse MNP were discussed. From this, the dependence of the FMMD signal on various factors such as the field amplitudes as well as the intrinsic particle core size distribution have been explored. It was found that FMMD signals strongly depend on the core size of the particles with strongest performance for large particles that remain limited to superparamagnetic nonhysteretic magnetization response, which limits core sizes to below 30 nm for iron oxide particles.

Considering biomedical applications, FMMD shows great promises in point‐of‐care diagnostic applications in magnetic biosensing and spectroscopy due to its highly sensitive reading of MNP signatures from also detecting intermodulation frequencies. Additionally, FMMD has further potential as an adjunct to form theranostic platforms with more established nanomedical techniques such as MPI and MFH because it offers frequency and amplitude combinations that are non‐interfering with MPI or MFH. Therefore, both methods could unfold their full potentials in uninterrupted combination while being supplemented by FMMD's additional information gain of MNP properties, concentration and binding state. Here, when it comes to materials characterization, FMMD provides a versatile tool for hydrodynamic, magnetic core size and size distribution detection as well as MNP quantification and novel low‐frequency scans were reviewed that offer a potentially fast and reliable way for MNP sizes and size distributions from a single non‐destructive measurement.

Concluding from the above, we consider FMMD's functional principles to be well understood, forming the basis for many promising applications. Nevertheless, much remains still to do for truly unlocking FMMD's full potential, be it as stand‐alone or adjunct / theranostic application in biomedicine: Key fields of improvement are a) optimizing instrumentation and physical FMMD setups to provide the ideal field parameters, which in turn correspond to b) the design of ideal MNP with properties maximizing the FMMD signal in application, and c) advancing theoretical modeling and predictive simulations to guide both instrumentation and MNP design along the way. Completing these tasks at hand, FMMD could be introduced as an adjunct to MPI for clinical diagnostics after receiving approved medical device status during clinical trials,^[^ ^271^ ^]^ potentially within the next 10 years, e.g. for vascular diagnostics.^[^ ^272^ ^]^ This work wishes to inspire such work in the future to advance dual‐frequency MNP spectroscopy in materials science and biomedicine from a fundamental and application‐driven approach alike.

## Conflict of Interest

The authors declare no conflict of interest.

## Supporting information



Supporting Information
