# Correction to “Fundamentals and Applications of Dual‐Frequency Magnetic Particle Spectroscopy: Review for Biomedicine and Materials Characterization”

**DOI:** 10.1002/advs.202513790

**Published:** 2025-09-06

**Authors:** Hans‐Joachim Krause, Ulrich M. Engelmann

**Affiliations:** ^1^ Institute of Biological Information Processing, Bioelectronics (IBI‐3) Forschungszentrum Jülich 52425 Jülich Germany; ^2^ Medical Engineering and Applied Mathematics FH Aachen University of Applied Sciences 52428 Jülich Germany

https://doi.org/10.1002/advs.202416838

In the originally published version of the paper, some equations contain typos and misleading references. We apologize for the inconvenience and wish the readers an undisturbed academic experience by stating the correct references and formulations as follows:

1. In the sentence directly following Equation (12), the effective field, ***H***_eff_, is defined subsequently in Equation (14), [not (13)].
2. In the paragraph directly following Equation (16), the Langevin function shall be referenced, cf. Equation (6) [not (5)].
3. Equation (21) shall be reading the 3rd derivative to ξ, i.e.: $\frac{d^{3}L\left(\xi \right)}{d\xi^{3}}=\frac{6}{\xi^{4}}-\frac{4\coth^{2}\xi }{\sinh^{2}\xi }-\frac{2}{\sinh^{4}\xi }\overset{\xi \to 0}{\to }\text{…}$
4. Equation (23) shall be reading the 5th derivative to ξ, i.e.: $\frac{d^{5}L\left(\xi \right)}{d\xi^{5}}=\frac{120}{\xi^{6}}-\frac{16\coth^{4}\xi }{\sinh^{2}\xi }-\frac{88\coth^{2}\xi }{\;\sinh^{4}\xi }-\frac{16}{\sinh^{6}\xi }\overset{\xi \to 0}{\to }\text{…}$
5. Equations (31), (32), and (33) shall all be reading only a scalar product after square brackets:
6. $m_{f_{1}+2f_{2}}=m_{p}\cdot \frac{2}{n}\sum_{i=0}^{n-1}L\left[\frac{m_{p}}{k_{B}T}\left(B_{0}+B_{1}\sin\left(2\pi f_{1}t_{i}\right)+B_{2}\sin\left(2\pi f_{2}t_{i}\right)\right)\right]\cdot \sin\left(2\pi \left(f_{1}+2f_{2}\right)t_{i}\right)$ (31)
7. $m_{f_{1}+3f_{2}}=m_{p}\cdot \frac{2}{n}\sum_{i=0}^{n-1}L\left[\frac{m_{p}}{k_{B}T}\left(B_{0}+B_{1}\sin\left(2\pi f_{1}t_{i}\right)+B_{2}\sin\left(2\pi f_{2}t_{i}\right)\right)\right]\cdot \cos\left(2\pi \left(f_{1}+3f_{2}\right)t_{i}\right)$ (32)
8. $m_{f_{1}+4f_{2}}=m_{p}\cdot \frac{2}{n}\sum_{i=0}^{n-1}L\left[\frac{m_{p}}{k_{B}T}\left(B_{0}+B_{1}\sin\left(2\pi f_{1}t_{i}\right)+B_{2}\sin\left(2\pi f_{2}t_{i}\right)\right)\right]\cdot \sin\left(2\pi \left(f_{1}+4f_{2}\right)t_{i}\right)$ (33)
9. In the paragraph following equations (31), (32), and (33), the switching from even to uneven functions can be seen from Equations (24)—(27) [not (28)–(23)].

