# Supplementary material for: Fundamentals and Applications of Dual‐Frequency Magnetic Particle Spectroscopy: Review for Biomedicine and Materials Characterization
Source: Adv Sci (Weinh). 2025 Feb 22;12(13):2416838. doi: 10.1002/advs.202416838 (PMC11967826; doi:10.1002/advs.202416838)
Supplement: Supplementary file 1 — Supporting Information [file ADVS-12-2416838-s001.pdf]

ADVANCED SCIENCE
Open Access

Supporting Information

for *Adv. Sci.*, DOI 10.1002/advs.202416838

Fundamentals and Applications of Dual-Frequency Magnetic Particle Spectroscopy: Review for Biomedicine and Materials Characterization

Hans-Joachim Krause and Ulrich M. Engelmann**

Supporting Information

Fundamentals and Applications of Dual-Frequency Magnetic Particle Spectroscopy: Review for Biomedicine and Materials Characterization

Hans-Joachim Krause and Ulrich M. Engelmann**

H.-J. Krause
Institute of Biological Information Processing, Bioelectronics (IBI-3), Forschungszentrum Jülich, 52425 Jülich, Germany
E-mail: h.-j.krause@fz-juelich.de

U. M. Engelmann
Medical Engineering and Applied Mathematics, FH Aachen University of Applied Sciences, 52428 Jülich, Germany
E-mail: engelmann@fh-aachen.de

A. Polydispersity Index

Instead of characterizing the width of the lognormal distribution by the dimensionless parameter σ, the quantity "polydispersity index" (PDI) is commonly used and defined by ISO22412:2017 hydrodynamic sizes analyzed with dynamic light scattering (DLS). The PDI refers to the hydrodynamic size distribution width assuming a Gaussian size distribution of particles, calculated from the second order fitting term of the exponential decay. In other words, it is defined as the square of the quotient of standard deviation and mean. PDI and σ are related by the following formulas:

$$\mathrm{PDI} = exp(\sigma^2) - 1 \qquad \text{and} \qquad \sigma = \sqrt{ln(\mathrm{PDI} + 1)}\ .$$

B. Verifying convergence of small and large amplitude calculations

In order to verify that the analytical small amplitude approximation of equation (28) and the numerical large amplitude calculation of equation (30) through (33) yield the same results in the limit of small excitation amplitudes, both calculations are compared in **Figure S1** for the second mixing term $f_1 + 2f_2$. At B_2 = 0.2 mT, the numerical calculation is about 1% lower than the analytical approximation, and almost 4% lower at B_2 = 0.5 mT. From the comparison one can conclude in first approximation that for excitation field amplitudes above 0.2 mT, the large amplitude calculation according to equation (30) through (33) should be utilized.

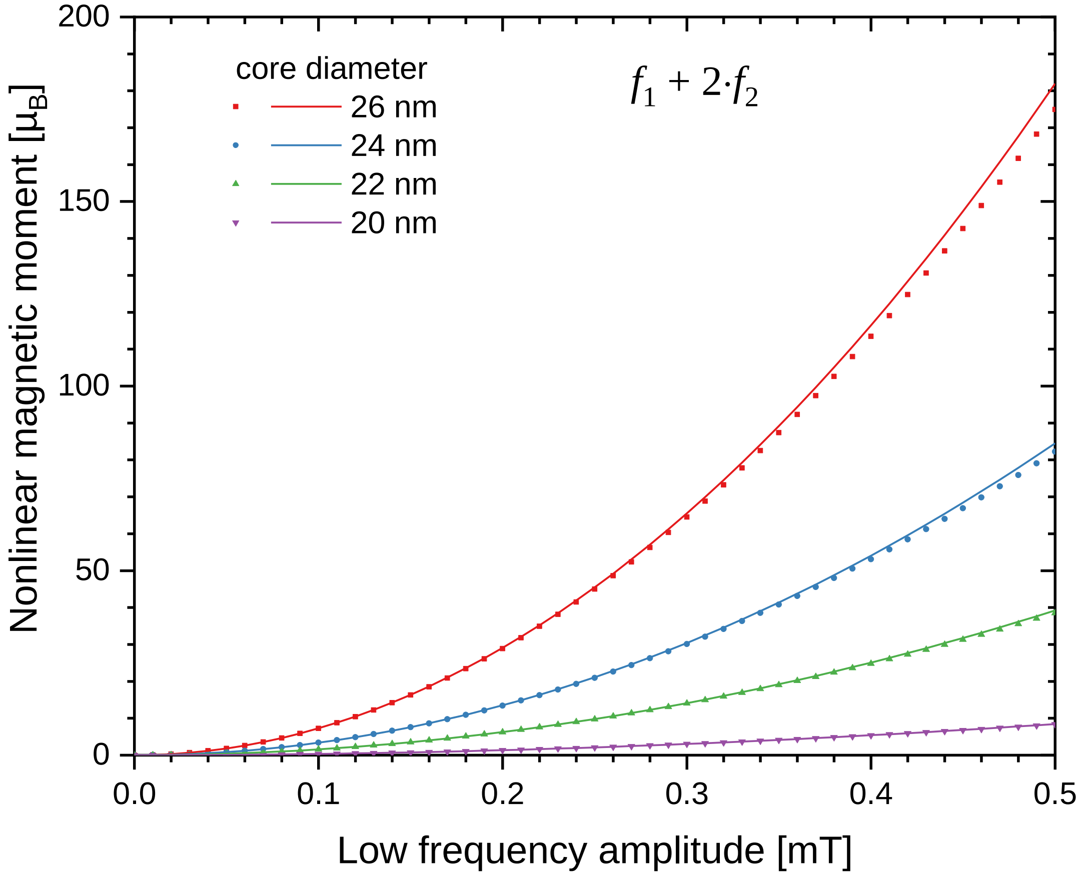

Figure S1. Comparison of the analytical small amplitude approximation (solid lines) and the numerical large amplitude calculation (symbols), for B_1 = 0.1 mT. The deviation is negligible for small low frequency excitations.

C. Scaling of frequency mixing responses with core size of MNP

Extracting the peak (i.e. maxima) values of FMMD signal generation leads to the size-dependent non-linear magnetic particle moments shown in **Figure S2**. With increasing MNP core size, the signal intensity increases by several orders of magnitude as expected and discussed above (cf. **Figure 5**).

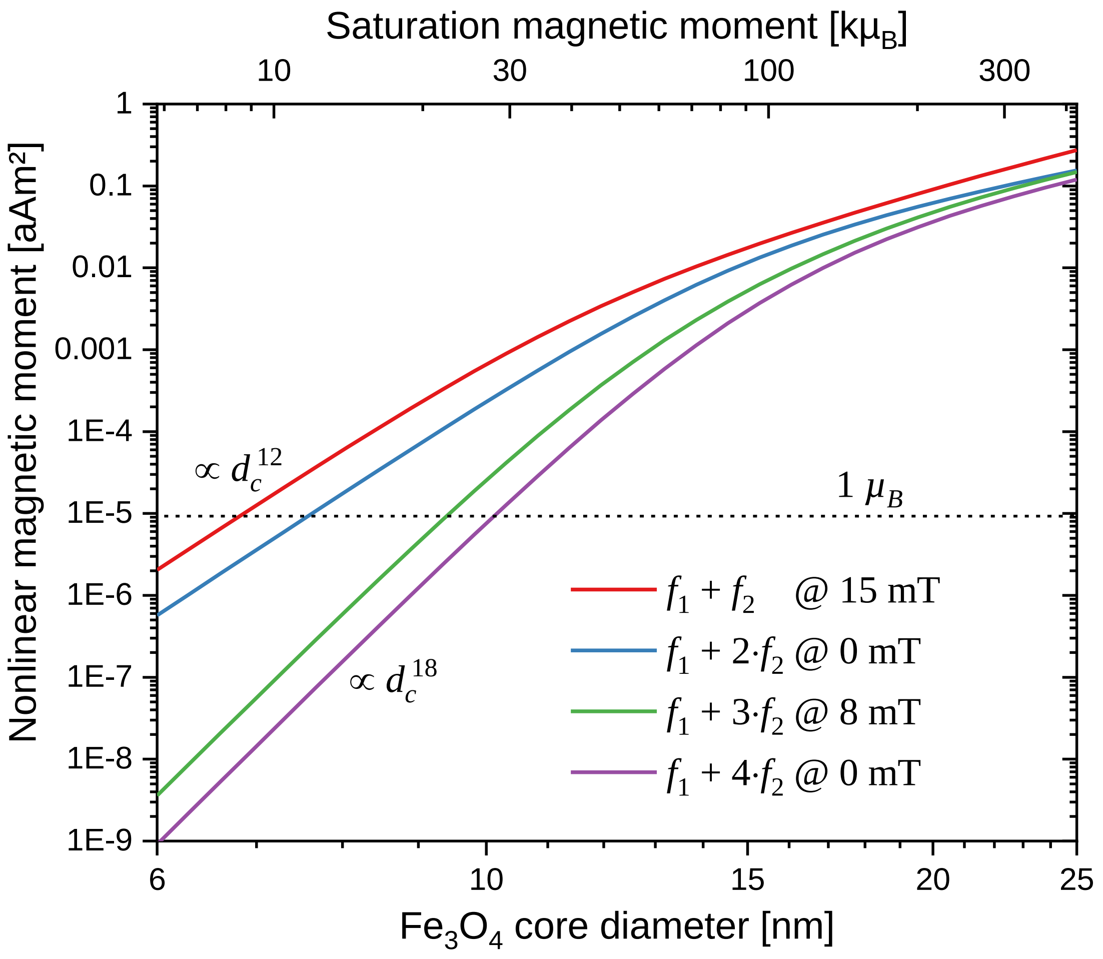

Figure S2. Peak of non-linear magnetic moment signals for the first four mixing frequencies in dependence of the particle size calculated for standard values of $B_1 = 1.29\ mT$ and $B_2 =$ 16.4 mT. The individually peaking offset fields are shown in the legend.

Interestingly, the nonlinear response signals of the mixing components $f_1 + f_2$ and $f_1 + 2 \cdot f_2$ scale with the fourth power of the saturation magnetic moment, $\propto m_p^4$, which corresponds according to equation (5) to the twelfth power of the particle core size, $\propto (d_c^3)^4 = d_c^{12}$. Note that this proportionality is expected for the $f_1 + 2 \cdot f_2$ frequency mixing component, as seen in the Taylor-based equation (28), 4th term. The $f_1 + f_2$ term exhibits the same scaling

because at fixed offset fields, the derivative of the Langevin function, $\mathcal{L}'' \propto m_p^4$ scales equally with the core diameter.

The higher mixing terms $f_1 + 3 \cdot f_2$ and $f_1 + 4 \cdot f_2$ scale even more pronounced with the particle's magnetic moment or core size, i.e. $\propto m_p^6 \propto d_c^{18}$. This directly illustrates that the generation of an FMMD signal is dominated by the largest size fraction of the probed MNP ensemble. The higher the harmonics, or the higher the mixing order, the steeper that size-dependent scaling. Such behavior of dominance excerted from large particles over the signal is well known for dynamic light scattering (DLS), where the signal intensity is governed by $I \propto d_H^6$, in accordance with Rayleigh scattering.[1]

Reference

[1] Berne B, Pecora R. Dynamic light scattering. USA, New York. 2000].
